# Noninvasive detection and prognostic stratification of biliary tract cancer using cell-free DNA fragmentomics: a model development and validation study

**DOI:** 10.1186/s43556-026-00468-7

**Published:** 2026-05-21

**Authors:** Jiwen Wang, Xiaojian Ni, Yuxuan Zheng, Song Wang, Hua Bao, Kun Fan, Sheng Shen, Dongqin Zhu, Qingxin Xie, Hairong Bao, Ruowei Yang, Chunyan Wang, Bohao Zheng, Shuang Chang, Xiuxiu Xu, Xiaoling Ni, Tao Suo, Xue Wu, Han Liu, Xiaokun Ma, Houbao Liu

**Affiliations:** 1 Department of Biliary Surgery, Zhongshan Hospital, Fudan University, Shanghai, 200032 China; 2 Biliary Tract Disease Center of Zhongshan Hospital, Fudan University, Shanghai, 200032 China; 3 Cancer Center, Zhongshan Hospital, Fudan University, Shanghai, 200032 China; 4 Biliary Tract Disease Institute, Fudan University, Shanghai, 200032 China; 5 Shanghai Engineering Research Center of Biliary Tract Minimal Invasive Surgery and Materials, Shanghai, 200032 China; 6 Department of General Surgery, Zhongshan-Xuhui Hospital, Fudan University, Shanghai, 200030 China; 7 Geneseeq Research Institute, Nanjing Geneseeq Technology Inc, Nanjing, 210032 China; 8 Department of Medical Oncology, The Third Affiliated Hospital of Sun Yat-Sen University, Guangzhou, 510630 China

**Keywords:** Biliary tract cancer, Early detection, Cell-free DNA, Liquid biopsy, Fragmentomics

## Abstract

Biliary tract cancer (BTC) is typically diagnosed at an advanced stage due to the lack of effective screening tools, resulting in limited therapeutic options and poor survival outcomes. Therefore, there is a critical need for non-invasive strategies that enable early detection and risk stratification. Fragmentomic profiling of cell-free DNA (cfDNA) captures genome-wide fragmentation patterns reflecting tumor-associated chromatin structure and genomic instability, providing a promising approach for non-invasive cancer detection. In this study, we developed a low-pass whole-genome sequencing (WGS)-based framework for BTC detection and postoperative risk assessment. Plasma samples were analyzed to derive three fragmentomic features, including copy number variation, fragment size distribution, and promoter fragmentation entropy, which were integrated into a machine-learning model trained using five-fold cross-validation. The model demonstrated robust performance across independent validation cohorts, outperforming individual fragmentomic features and conventional serum biomarkers, and accurately distinguished BTC from benign biliary diseases. Longitudinal analyses revealed that cfDNA fragmentomic risk scores dynamically tracked disease burden and treatment response. Importantly, postoperative risk scores were independently associated with disease-free survival, highlighting their prognostic value. Collectively, these findings establish a scalable and cost-effective framework for cfDNA-based BTC detection and monitoring using low-pass WGS data. This approach shows strong potential for targeted screening in high-risk populations and for guiding personalized postoperative surveillance and clinical management for BTC patients.

**Supplementary Information:**

The online version contains supplementary material available at 10.1186/s43556-026-00468-7.

## Introduction

Biliary tract cancer (BTC) is a heterogeneous group of aggressive malignancies arising from the biliary epithelium, including gallbladder carcinoma (GBC) and cholangiocarcinoma (CCA) [1–5]. CCA is further subdivided into intrahepatic (iCCA), perihilar (pCCA), and distal (dCCA) subtypes, each with distinct anatomical locations, clinical behaviors, and molecular characteristics. Although BTC is relatively uncommon, recent epidemiological analyses indicate that its absolute global burden has increased substantially, with incident cases and deaths rising over recent decades due to population growth and aging [6–8]. In particular, iCCA has shown increasing mortality trends in many regions, and overall BTC prognosis remains poor, with most patients diagnosed at advanced stages when curative surgery is no longer feasible. These epidemiological and clinical features underscore the urgent need for effective strategies for early detection and risk stratification across BTC subtypes.

Current diagnostic approaches for BTC mainly rely on imaging, endoscopic procedures, and serum biomarkers such as carbohydrate antigen 19**-**9 (CA19**-**9) and carcinoembryonic antigen (CEA). However, these methods have notable limitations. Imaging techniques, such as computed tomography (CT) and magnetic resonance imaging (MRI), often fail to detect small or early-stage lesions and cannot reliably distinguish malignant from benign biliary strictures. Endoscopic cytology, although specific, suffers from low sensitivity, while CA19**-**9 and CEA lack sufficient diagnostic accuracy, particularly in early-stage disease [9–12]. Moreover, CA19**-**9 levels can also be elevated in benign biliary inflammation or obstruction, as well as other gastrointestinal malignancies [2]. Therefore, there is a critical need for non-invasive biomarkers with higher sensitivity and specificity for BTC detection and monitoring.

Circulating cell-free DNA (cfDNA) fragmentomics, which analyzes genome-wide fragmentation patterns shaped by chromatin structure and tumor-associated genomic instability, has emerged as a promising approach for non-invasive cancer detection. Fragmentomic features, such as fragment end motifs [13], fragment size ratio [14], copy number variations (CNV), nucleosome positioning footprints [15], and DNA methylation patterns [16], have shown potential for improving cancer detection across multiple cancer types. However, tumor-derived cfDNA signals are often obscured by abundant background DNA from non-malignant hematopoietic cells, thereby limiting sensitivity. Promoter fragmentation entropy (PFE) has been proposed as a complementary feature that captures fragmentation patterns in gene promoter regions and correlates with gene expression [17–19]. Nonetheless, individual fragmentomic markers may still lack sufficient diagnostic accuracy, and their combined value has not been comprehensively evaluated in BTC. Integrating multiple fragmentomic features within a machine-learning framework may enhance diagnostic performance and provide additional value for disease monitoring.

To address these challenges, we conducted DECIPHER-CHOL (Detecting Early Cancer by Inspecting ctDNA Features in cholangiocarcinoma), a multi-cohort diagnostic study designed to develop and validate a cfDNA fragmentomics-based approach for BTC detection and postoperative risk stratification. We hypothesized that integrating multiple cfDNA fragmentomic features would outperform single-feature models and conventional serum biomarkers for BTC detection. We therefore built an ensemble machine-learning model based on copy number variation, fragment size distribution, and promoter fragmentation entropy derived from low-depth plasma whole-genome sequencing (WGS) and validated its diagnostic performance in independent cohorts. Postoperative fragmentomic risk scores independently predicted disease-free survival, supporting their potential for surveillance and personalized management.

## Results

### Participant characteristics

**Fig. 1:**
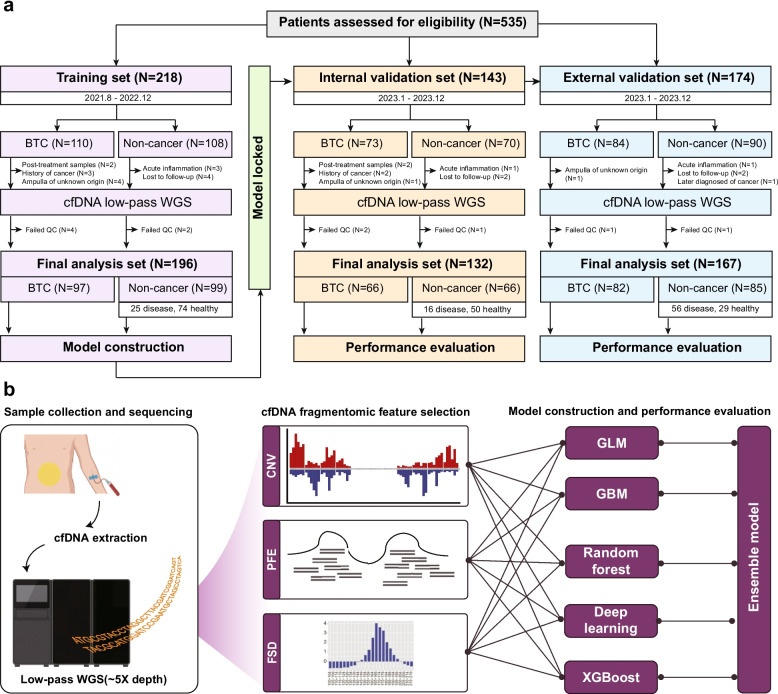
Study design and model development workflow. **a** Flowchart showing patient inclusion and cohort distribution for model training (training set) and performance evaluation (internal and external validation sets). (**b**) Schematic representation of the model development process, integrating three cfDNA fragmentomic features with five machine learning algorithms. The schematic was created by the authors with the assistance of an AI-based graphic design tool (Nano Banana Pro). BTC, biliary tract cancer; WGS, whole-genome sequencing; cfDNA, cell-free DNA; CNV, copy number variation; FSD, fragment size distribution; PFE, promoter fragmentation entropy; GLM, generalized linear model; GBM, gradient boosting machine

This multi-center study initially enrolled 535 participants, including 267 patients diagnosed with BTC and 268 non-cancer individuals (Fig. 1a). The training cohort was exclusively used for model construction, whereas the internal and external validation datasets were withheld until the final model evaluation. The baseline characteristics of the participants are summarized in Table 1. In the training cohort, the median age was 59 years (range: 45–75 years) for non-cancer individuals and 67 years (range: 38–83 years) for BTC patients. A slightly higher proportion of males was observed for both groups. Among BTC patients, 81.4% were diagnosed at stage I/II. The predominant anatomical subtype was dCCA at 47.4% (46/97), followed by GBC (33.1%; 32/97) and pCCA (16.4%; 16/97), while iCCA cases were infrequent. All but three BTC patients had adenocarcinoma histology. In terms of tumor differentiation, 40.2% (39/97) were poorly differentiated, 52.6% (51/97) were moderately differentiated, and 2.0% (2/97) were well differentiated. Non-cancer patients were further categorized into those with benign biliary tract conditions and healthy individuals without known diseases. Among them, 74.7% (74/97) were asymptomatic individuals voluntarily enrolled through routine physical examinations, while the remaining 25.3% (25/97) had non-malignant biliary tract diseases, such as benign biliary stricture, gallbladder adenomyomas, and gallbladder polyps.

**Table 1:** Clinical characteristics of participants

	**Training**		**Internal validation**		**External validation**	
**Characteristic**	**Non-cancer (*N* = 99)**	**BTC (*N* = 97)**	**Non-cancer (*N* = 66)**	**BTC (*N* = 66)**	**Non-cancer (*N* = 85)**	**BTC (*N* = 82)**
**Age**						
Median (range)	59 (45,75)	67 (38,83)	57 (75,73)	65 (38,84)	56 (46,74)	63 (39,74)
**Sex**						
Male	57 (57.6%)	55 (56.7%)	38 (57.6%)	38 (57.6%)	42 (49.4%)	46 (56.1%)
Female	42 (42.4%)	42 (43.3%)	28 (42.4%)	28 (42.4%)	43 (50.6%)	36 (43.9%)
**Stage**						
I	-	14 (14.4%)	-	10 (15.2%)	-	16 (19.5%)
II	-	65 (67.0%)	-	46 (69.6%)	-	47 (57.3%)
III	-	18 (18.6%)	-	10 (15.2%)	-	19 (23.2%)
**Anatomical type**						
iCCA	-	3 (3.1%)	-	0 (0.0%)	-	22 (26.8%)
pCCA	-	16 (16.4%)	-	21 (31.9%)	-	13 (15.9%)
dCCA	-	46 (47.4%)	-	22 (33.3%)	-	30 (36.6%)
GBC	-	32 (33.1%)	-	23 (34.8%)	-	17 (20.7%)
**Histology**						
Adenocarcinoma	-	94 (96.9%)	-	64 (96.9%)	-	75 (91.5%)
Adenosquamous carcinoma	-	3 (3.1%)	-	2 (3.1%)	-	7 (8.5%)
**Differentiation**						
Poor	-	40 (41.2%)	-	20 (30.2%)	-	47 (57.3%)
Moderate	-	50 (51.5%)	-	44 (66.7%)	-	30 (36.6%)
Well	-	2 (2.1%)	-	0 (0.0%)	-	0 (0.0%)
Unknown	-	5 (5.2%)	-	2 (3.1%)	-	5 (6.1%)
**Non-cancer subgroup**						
Disease	25 (25.3%)	-	16 (24.2%)	-	56 (65.9%)	-
Healthy	74 (74.7%)	-	50 (75.8%)	-	29 (34.1%)	-
**Benign disease type**						
Ployp	17 (68.0%)	~	10 (62.5)	~	38 (67.9%)	~
Stone	8 (32.0%)	~	6 (37.5%)	~	18 (32.1%)	~
**WBC (10**^**9**^**/L)**						
Median (range)	5.70 (3.20, 9.50)	5.45 (2.15, 10.9)	6.19 (3.50, 9.90)	5.50 (2.88, 8.57)	5.70 (3.28, 9.60)	6.26 (3.31, 11.1)
> 10	0 (0%)	2 (2.1%)	0 (0%)	0 (0%)	0 (0%)	3 (3.7%)
≤ 10	99 (100%)	95 (97.9%)	66 (100%)	66 (100%)	85 (100%)	79 (96.3%)
**DB (µmol/L)**						
Median (range)	4.51 (1.70, 6.79)	16.8 (0.80, 372)	4.09 (1.71, 6.68)	15.1 (1.10, 294)	4.13 (1.74, 6.66)	22.2 (0.900, 93.0)
> 6.8	0 (0%)	61 (62.9%)	0 (0%)	36 (54.5%)	0 (0%)	54 (65.9%)
≤ 6.8	99 (100%)	36 (37.1%)	66 (100%)	30 (45.5%)	85 (100%)	28 (34.1%)
**Max tumor diameter (cm)**						
Median (range)	~	2.00 (0.600, 9.50)	~	2.00 (0.600, 7.00)	~	2.50 (0.700, 11.5)

*GBC* Gallabladder carcinoma, *iCCA* intrahepatic cholangiocarcinoma, *pCCA* perihilar cholangiocarcinoma, *dCCA* distal cholangiocarcinoma, *WBC* White blood cell, *DB* Direct bilirubin

Participant characteristics in the validation cohorts were generally comparable. However, the independent external validation set exhibited notable differences, including a higher proportion of iCCA (26.8%) and poorly differentiated tumors (57.3%) among BTC patients. Moreover, unlike the internal validation set, the external cohort comprised a larger proportion of individuals with benign biliary tract diseases compared to healthy participants (65.9% vs. 34.1%). These differences highlight the heterogeneity of the external dataset, thereby serving as a complementary cohort that enhances the generalizability and robustness of the model across diverse clinical scenarios.

### cfDNA fragmentomic feature profiles

We systematically evaluated these three cfDNA fragmentomic features, namely copy number variation (CNV), fragment size distribution (FSD), and promoter fragmentation entropy (PFE), for their ability to distinguish BTC patients from non-cancer participants. First, genome-wide CNV analysis revealed notable chromosomal alterations in BTC patients, particularly among those with more advanced-stage diseases. Recurrent gains were observed on chromosomal arms 1q, 5p, and 6q, whereas losses were detected on 4p, 6p, and 8p (Fig. S1a, b). These alterations are consistent with tumor-associated genomic instability and were more prominent in later clinical stages.

Beyond large-scale chromosomal alterations, we next examined cfDNA fragment size distribution. BTC patients exhibited significantly higher normalized coverage of short cfDNA fragments (100-150 bp) compared with non-cancer controls across all chromosome arms (Fig. S2a, b). Trend analysis across clinical stages demonstrated a significant monotonic increase in short fragment enrichment from stage I to stage III across all examined fragment size bins within 100-150 bp (Jonckheere-Terpstra test, all *p* < 0.001) (Table S1). These findings support an association between altered cfDNA fragmentation patterns and increasing tumor burden.

We further assessed fragmentation complexity at the promoter level, characterized by the PFE feature. Based on the 1,203 selected PFE features, we derived an integrated PFE score, which was significantly higher in BTC patients compared with non-cancer controls (Wilcoxon, *p* < 0.001; effect size: 0.784; 95% CI: 0.736—0.823) (Fig. S3a). Consistently, normalized PFE values demonstrated a similar pattern of elevated entropy in BTC across both the training and internal validation cohorts (Fig. S3b, c), indicating robust cross-cohort reproducibility. We also performed a depth-stability analysis by downsampling 50 × composite samples to 40x, 30x, 20x, 10x, and 5x. Even at 5 × coverage, PFE profiles remained highly correlated with their corresponding 50 × profiles (Pearson correlation coefficient > 0.9 across samples) (Fig. S3d), supporting the robustness of entropy-based fragmentation patterns at the sequencing depth used in this study. To explore the biological relevance of the PFE feature, KEGG and GO enrichment analyses revealed significant involvement in pathways related to protein and amino acid metabolism, small RNA processing, and complement component C3b binding (Fig. S3e), suggesting that entropy-based promoter fragmentation patterns may reflect biologically meaningful tumor-associated processes. Collectively, these results demonstrate that each cfDNA fragmentomic feature captures distinct tumor-associated signals capable of discriminating BTC from non-cancer individuals.

### Evaluating model performance

**Fig. 2:**
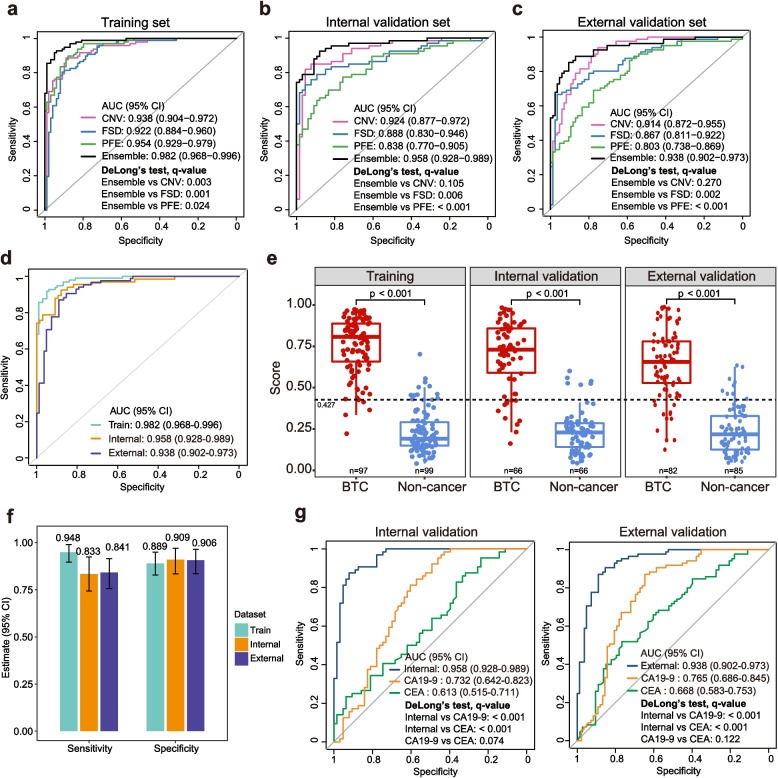
Model performance and comparison to conventional serum biomarkers. **a-c** Receiver operating characteristic (ROC) curves evaluating the diagnostic performance of individual fragmentomic features (CNV, FSD, and PFE) and the integrated model in the training set (**a**), internal validation set (**b**), and external validation set (**c**). **d** ROC curves of the ensemble model across the training, internal validation, and external validation cohorts. **e** Distribution of risk scores for BTC and non-cancer participants in each dataset, with the dotted line indicating the cutoff corresponding to 95% sensitivity derived from the training set. **f** Sensitivity and specificity of the ensemble model at the predefined cancer prediction cutoff across all datasets. **g** ROC curves comparing the performance of the ensemble model with conventional serum biomarkers (CEA and CA19**-**9) in the internal and external validation sets. CNV, copy number variation; FSP, fragment size distribution; PFE, promoter fragmentation entropy; AUC, area under the curve; CI, confidence interval; CEA, carcinoembryonic antigen; CA19**-**9, carbohydrate antigen 19**-**9

We first evaluated the discriminative performance of each cfDNA fragmentomic feature individually (Fig. 2a-c). In the training set, PFE achieved the highest AUC among single-feature models (0.954, 95% CI: 0.929–0.979), followed by CNV (0.938, 95% CI: 0.904–0.972) and FSD (0.922, 95% CI: 0.884–0.960). In both the internal and external validation sets, CNV was the best-performing individual feature. We then integrated CNV, PFE, and FSD into an ensemble model, which achieved AUCs of 0.982 (95% CI: 0.968–0.996) in the training set, 0.958 (95% CI: 0.928–0.989) in the internal validation set, and 0.938 (95% CI: 0.902–0.973) in the external validation set (Fig. 1b; 2a-d). Compared with individual-feature models, the ensemble model showed significant improvement over CNV in the training set (DeLong’s test, q = 0.003). Although the improvement over CNV did not reach statistical significance in the validation cohorts, the ensemble model consistently outperformed both FSD and PFE in the internal (q = 0.006 and < 0.001) and external (q = 0.002 and < 0.001) validation datasets.

Model-derived risk scores were significantly higher in cancer compared with non-cancer participants across all datasets (Wilcoxon test, *P* < 0.001) (Fig. 2e; Table S2). To prioritize early detection and minimize missed cases, we selected a cutoff of 0.427, corresponding to 95% sensitivity in the training set. At this threshold, the model maintained high specificity of 0.889 (95% CI: 0.810–0.943), 0.909 (95% CI: 0.813–0.966), and 0.906 (95% CI: 0.823–0.959) in the training, internal, and external validation sets, respectively, with correspondingly high PPV and NPV within each cohort (Fig. 2f; Table 2). Model performance was also evaluated at a cutoff corresponding to 95% specificity in the training set for reference (Table S3). It is important to note that PPV and NPV are prevalence-dependent and vary across clinical contexts. Based on the observed sensitivity and specificity, PPV would be low in general screening populations with very low disease prevalence (0.8%–8.5% at 0.1%–1% prevalence) but would increase markedly in higher-risk or clinically suspected populations (~50% at 10% prevalence, ~ 70% at 20% prevalence) (Table S4). In contrast, NPV remains consistently high across a broad range of prevalence levels. These prevalence-dependent characteristics suggest that the model may be particularly useful for ruling out disease in low-prevalence settings and for prioritizing individuals for further diagnostic evaluation in higher-risk clinical populations.

**Table 2:** Performance metrics across datasets

Cutoff = 0.427 corresponds to 95% sensitivity in the training set							
		Training		Internal validation		External validation	
		Actual		Actual		Actual	
		BTC	Non-cancer	BTC	Non-cancer	BTC	Non-cancer
Predict	BTC	92	11	55	6	69	8
	Non-cancer	5	88	11	60	13	77
Sensitivity (95% CI)		0.949 (0.884—0.983)		0.833 (0.721—0.914)		0.842 (0.744—0.913)	
Specificity (95% CI)		0.889 (0.810—0.943)		0.909 (0.813—0.966)		0.906 (0.823—0.959)	
PPV (95% CI)		0.893 (0.817—0.946)		0.902 (0.798—0.963)		0.896 (0.806—0.954)	
NPV (95% CI)		0.946 (0.879—0.982)		0.845 (0.740—0.920)		0.856 (0.766—0.921)	
Accuracy (95% CI)		0.918 (0.871—0.953)		0.871 (0.802—0.923)		0.874 (0.814—0.920)	

Calibration analyses demonstrated overall agreement between predicted probabilities and observed outcomes across cohorts (Fig. S4). Calibration was most accurate in the low- to moderate-risk range, with minor deviation from ideal calibration observed at the highest predicted risk levels. Brier scores were 0.079 in the training cohort, 0.104 in the internal validation cohort, and 0.126 in the external validation cohort, indicating low overall prediction error. Calibration slopes exceeded 1 across cohorts, suggesting deviation from ideal calibration, particularly at the extremes of predicted risk.

Conventional serum biomarkers were also evaluated in comparison with our ensemble model. CEA showed limited performance in distinguishing BTC patients from non-cancer participants, with AUCs of 0.613 and 0.668 in the internal and external validation sets, respectively (Fig. 2g). CA19**-**9 performed slightly better than CEA in both datasets, although the differences were not statistically significant (DeLong’s test, q = 0.074 and 0.122). Across cohorts, model-derived prediction scores were positively correlated with both biomarkers, with Spearman correlation coefficients ranging from 0.28 to 0.50 (all *p* < 0.001), indicating a modest association (Fig. S5). Notably, integrating the ensemble model with CEA and/or CA19**-**9 did not significantly improve predictive performance over the model alone in any cohort (DeLong’s test, q > 0.05) (Fig. S6). Collectively, these findings suggest that the ensemble model captured tumor-derived signals beyond those identified by individual cfDNA features or conventional serum biomarkers, with limited added benefit from combining with serum biomarkers.

### Performance evaluation across various subgroups

**Fig. 3:**
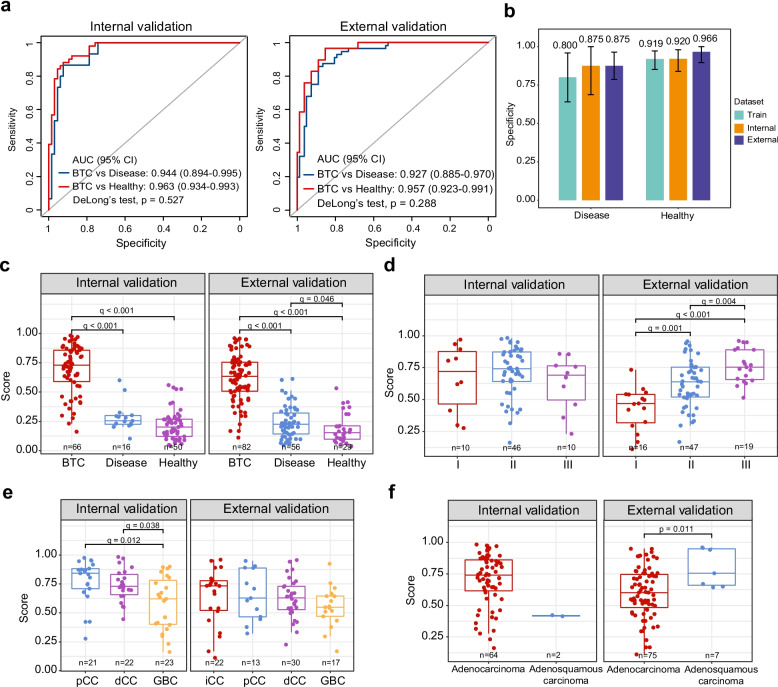
Model performance across clinicopathological subgroups. **a** Receiver operating characteristic (ROC) curves showing the model’s ability to discriminate BTC patients from non-cancer controls (stratified into healthy individuals and those with benign biliary disease) in the internal (left) and external (right) validation cohorts. **b** Specificity of the ensemble model for non-cancer subgroups at the predefined cutoff corresponding to 95% sensitivity. **c-f** Distribution of risk scores in validation sets, stratified by disease status (BTC, disease, and healthy) (**c**), clinical stage (**d**), anatomical tumor location (**e**), and histological subtype (**f**). iCCA, intrahepatic cholangiocarcinoma; pCCA, perihilar cholangiocarcinoma; dCCA, distal cholangiocarcinoma; GBC, gallbladder cancer

We assessed the model’s performance across different clinical scenarios to evaluate its robustness. Non-cancer participants may present with benign biliary tract diseases, which are often challenging to distinguish from malignancies. In the internal validation set, individuals with benign diseases comprised 24.2% of the non-cancer group, whereas this proportion was nearly three times higher in the external validation set (Table 1). In both cohorts, the ensemble model demonstrated strong discrimination for BTC versus disease controls and BTC versus healthy controls, with AUCs of 0.944 and 0.963 in the internal set (DeLong’s test, *p* = 0.527) and 0.927 and 0.957 in the external set (DeLong’s test, *p* = 0.288) (Fig. 3a, b). Using the high-sensitivity cutoff, specificity remained above 0.80 and reached 0.966 for healthy participants in the external cohort.

To further evaluate performance in clinically challenging scenarios, we examined stage-stratified BTC versus benign disease controls. Even for stage I BTC, the most diagnostically ambiguous subset, the ensemble model achieved high discriminative performance, with AUCs of 0.919 (95% CI: 0.811–1.000) and 0.800 (95% CI: 0.666–0.934) in the internal and external cohorts, respectively (Fig. S7a-c). At the predefined cutoff, specificity against benign controls was 0.875 in both cohorts (Table S5). Early-stage BTC samples also exhibited significantly higher prediction scores than benign cases (Wilcoxon test, q < 0.001) (Fig. S7d-f; Table S2), highlighting the model’s potential utility in real-world diagnostic settings.

We next examined the distribution of risk scores across clinical subgroups. In both validation sets, risk scores were significantly higher in BTC compared with benign disease and healthy controls (Wilcoxon, q < 0.001) (Fig. 3c; Table S2). In the external validation set, benign disease also showed slightly higher scores than healthy controls (Wilcoxon, q = 0.046), whereas this difference was not significant in the internal set. When stratified by stage, the external validation set exhibited a clear increasing trend, with significant pairwise differences (Fig. 3d). In contrast, stage-associated differences were less pronounced in the internal validation set, likely reflecting limited and imbalanced sample sizes.

For anatomical subsites, the internal validation set showed modest but significant differences (pCCA vs GBC: q = 0.012; dCCA vs GBC: q = 0.038), whereas similar subsite differences were not observed in the external cohort (Fig. 3e). To assess whether these apparent differences were confounded by stage distribution, we performed stage-stratified analyses. Stacked bar plots revealed that the distribution of BTC subsites varied across stages within and between cohorts (Fig. S8a). In stage-stratified comparisons, risk score differences between subsites were generally inconsistent and often limited by small subgroup sizes (Fig. S8b-d). These results suggest that the apparent subsite differences seen in unstratified analyses are likely influenced by cohort-specific stage and subsite distributions rather than reflecting stable biological differences across BTC subsites.

Regarding histology, adenosquamous carcinoma had higher scores than adenocarcinoma in the external validation set (Wilcoxon, *p* = 0.011), but this comparison was not evaluable in the internal set due to very small numbers (Fig. 3f). No significant difference was observed between poorly and moderately differentiated tumors (Fig. S9), though whether this reflects a true lack of association or limited statistical power requires further investigation. Collectively, these results indicate that the model reliably discriminates BTC from non-cancer controls, while stage- and subtype-associated variations appear cohort-dependent and warrant cautious interpretation.

### Risk stratification and disease monitoring based on the BTC risk score

**Fig. 4:**
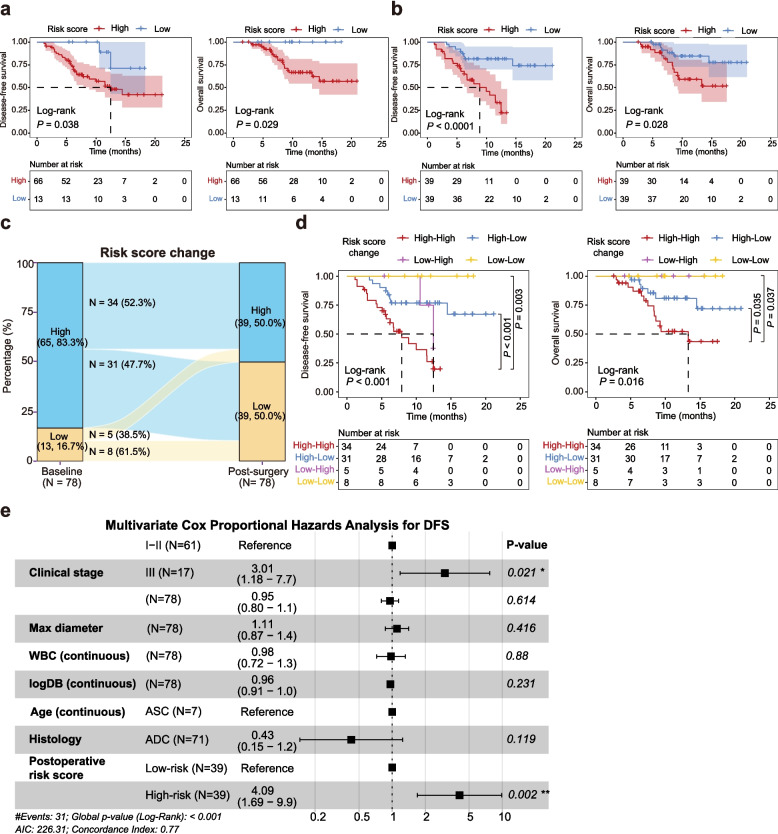
CfDNA-based risk scores and their association with patient survival in BTC. **a** Kaplan–Meier curves for disease-free survival (DFS) and overall survival (OS) based on risk scores derived from preoperative plasma samples. **b** Kaplan–Meier curves for DFS and OS based on risk scores derived from postoperative plasma samples. **c** Sankey plot illustrating individual transitions in risk status from pre- to post-surgical time points among patients with paired samples. **d** Kaplan–Meier curves showing DFS and OS of BTC patients according to dynamic changes in risk scores across paired samples. **e** Forest plot showing hazard ratios (HRs) with 95% confidence intervals (CIs) for key clinical covariates and postoperative risk scores in multivariate Cox regression analysis

Among the 82 cancer patients in the external validation cohort, 79 had available survival data, and 78 had plasma samples collected within three months after surgery (75 within one week) for BTC risk score analysis (Table S6). Patients classified as high risk based on baseline plasma samples exhibited significantly shorter disease-free survival (DFS) and overall survival (OS) compared with those in the low-risk group (*P* = 0.038 and *P* = 0.029, respectively) (Fig. 4a). This survival disadvantage was even more pronounced when postoperative plasma samples were used for risk stratification (*P* < 0.0001 for DFS and *P* = 0.028 for OS) (Fig. 4b). Among the 78 patients with paired pre- and post-operative samples, individuals were stratified into four groups according to changes in risk scores: high-high (*N* = 34, 52.3%), high-low (*N* = 31, 47.7%), low–high (*N* = 5, 38.5%), and low-low (*N* = 8, 61.5%) (Fig. 4c). As anticipated, patients with persistently high risk scores had the poorest DFS and OS outcomes (Fig. 4d). Notably, their survival was significantly worse than that of patients whose scores decreased from high to low following surgery (*P* < 0.001 for DFS and *P* = 0.035 for OS), suggesting that dynamic changes in cfDNA-based risk scores may reflect postoperative residual disease burden and patient prognosis.

We then evaluated the prognostic value of the BTC risk score measured in postoperative blood samples. In the external postoperative BTC cohort (*N* = 78), univariate Cox analyses identified advanced stage, adenosquamous histology, poor differentiation, larger tumor diameter, and a high postoperative risk score as predictors of shorter DFS (Table S7). The postoperative risk score was strongly associated with DFS, both when dichotomized using the diagnostic cutoff (HR 4.772, 95% CI 2.038%–11.173%; *P* < 0.001) and when analyzed as a continuous variable (HR 11.226, 95% CI 2.314%–54.450%; *P* = 0.003). In contrast, the baseline risk score showed only a borderline association when dichotomized (HR 4.060, 95% CI 0.967%–17.056%; *P* = 0.056).

To account for potential confounding while maintaining model stability given the number of events per variable and sparse subgroups, we prespecified a parsimonious multivariable Cox model adjusting for age, clinical stage (I–II vs III), tumor burden (maximum diameter), histology (ADC vs ASC), and baseline inflammatory/cholestatic markers (WBC and direct bilirubin [DB]). In this primary adjusted model, a high postoperative risk score remained independently associated with worse DFS (HR 4.09, 95% CI 1.69–9.90; *P* = 0.002), whereas baseline risk score was not significant (Fig. 4e; Fig. S10). Sensitivity analyses confirmed the robustness of postoperative risk score as an independent predictor after additional adjustment for postoperative complications (Fig. S11a), tumor subsite (stratified Cox; Fig. S11b), alternative stage coding (I/II/III; Fig. S11c), categorization of WBC/DB (Fig. S11d), and restriction to patients sampled within one week after surgery (*N* = 75; Fig. S11e). Across all models, postoperative risk score effect estimates were consistent and statistically significant.

Finally, multicollinearity and model assumptions were evaluated for the primary and sensitivity models. No concerning multicollinearity was observed (all VIFs < 5), and the proportional hazards assumption was satisfied based on Schoenfeld residual tests (all *P* > 0.05) (Tables S8, S9). Collectively, these results support the postoperative risk score as a robust and independent predictor of DFS in BTC.

## Discussion

Early detection of BTC remains a critical clinical challenge due to its typically late diagnosis and lack of effective screening tools. Our study provides compelling evidence that integrating multiple cfDNA fragmentomic features enables robust detection of BTC through a minimally invasive liquid biopsy. The assay is most informative in higher-risk populations, supporting its use as a triage tool to prioritize patients for further diagnostic evaluation. Beyond detection, postoperative cfDNA-based risk scores may aid in stratifying patient survival and monitoring minimal residual disease (MRD), highlighting broader clinical applications. These findings lay a foundation for future studies aimed at risk-adapted patient management and early intervention.

While early detection strategies for hepatocellular carcinoma (HCC) have been relatively well established, supported by defined high-risk populations, screening guidelines, and serum biomarkers such as alpha-fetoprotein (AFP) combined with imaging, BTC lacks such clear frameworks [20–22]. The absence of standardized screening protocols and reliable biomarkers, coupled with BTC’s anatomical complexity and biological heterogeneity [2, 23], contributes to ongoing challenges in early diagnosis. From a biological perspective, tumor-derived cfDNA exhibits distinct fragmentation patterns shaped by chromatin accessibility, nucleosome positioning, and cell death processes. Genome-wide fragmentomic profiling captures these patterns, enabling a comprehensive molecular signature that reflects tumor presence and burden beyond single-marker approaches. Importantly, although ultra-deep sequencing may maximize concordance between PFE and mRNA abundance, our analyses demonstrate that PFE features remain highly stable even at 5 × coverage, indicating that the observed cancer-associated signals are unlikely to be dominated by stochastic noise or prior assumptions. Moreover, biliary tract cancers are consistently reported as among the most detectable cancer types in multi-cancer early detection studies [24, 25], suggesting that fragmentation-based signals in this cancer type are relatively pronounced. Consistent with our analyses, BTC samples exhibited significantly higher PFE values compared with non-cancer controls, supporting that these signals are robust and discriminative. Therefore, even if the correlation between PFE and mRNA expression is attenuated at low sequencing depth, the observed fragmentation patterns remain sufficient for reliable cancer versus non-cancer discrimination. The consistent and robust performance of our model across internal and external cohorts highlights its potential to fill the significant gap in BTC early screening.

A key strength of our cfDNA fragmentomics model is its robust ability to distinguish BTC not only from healthy individuals but also from patients with benign biliary diseases, which often mimic malignancy and pose significant diagnostic challenges. In both validation cohorts, the ensemble model demonstrated strong discrimination for BTC versus disease controls and BTC versus healthy controls, with AUCs of 0.944 and 0.963 in the internal validation set and 0.927 and 0.957 in the external validation set, respectively. Notably, even stage I BTC, arguably the most diagnostically challenging subgroup, remained distinguishable from benign controls, supporting the potential clinical utility of this approach in early-stage disease. Compared with conventional serum biomarkers, including CEA and CA19-9, the ensemble model achieved superior and more consistent performance, and integration with these biomarkers did not significantly improve discrimination. Model-derived risk scores were significantly higher in BTC than in non-cancer controls across cohorts and showed an increasing trend with advancing stage in the external validation set, suggesting an association with tumor burden. Although differences across anatomical subsites and histological subtypes were observed in certain cohorts, these patterns were inconsistent and appeared influenced by stage distribution and sample size, warranting cautious interpretation. Collectively, these findings indicate that the model captures robust tumor-derived signals beyond conventional biomarkers and may aid in differentiating malignancy from benign biliary pathology, a clinically relevant scenario that often leads to diagnostic uncertainty and potentially unnecessary invasive procedures.

Furthermore, our study demonstrates that cfDNA fragmentomic risk scores, derived from both pre- and post-operative plasma samples, were significantly associated with DFS and OS in BTC patients, with higher scores correlating with poorer outcomes. This highlights the prognostic value of cfDNA fragmentation patterns beyond their diagnostic utility, reflecting tumor aggressiveness and recurrence risk. Notably, the post-surgical risk score exhibited stronger prognostic associations than the pre-surgical score, suggesting the potential presence of MRD, that is, microscopic tumor deposits remaining after surgical resection undetectable by conventional imaging or histopathology. Thus, cfDNA fragmentomic signatures in post-operative plasma may serve as a molecular indicator of MRD, enabling earlier identification of patients at elevated risk of relapse. Indeed, these observations align with accumulating evidence supporting liquid biopsy for MRD detection and treatment monitoring across diverse cancer types [26–30].

Importantly, although our model was initially developed using pre-operative samples to differentiate malignant from non-malignant cases, it retained predictive ability for patient outcomes when applied to postoperative samples. This cross-context applicability suggests that the model captures stable and biologically meaningful cfDNA features that transcend temporal sampling. The ability of a baseline-trained model to retain prognostic relevance post-surgery not only underscores its robustness but also expands its clinical utility, offering a non-invasive platform for both early diagnosis and longitudinal disease monitoring. As such, this approach may help bridge the current gap between static diagnostic tools and dynamic surveillance strategies in BTC care. Collectively, these results position cfDNA fragmentomic profiling as a promising multi-faceted tool supporting early detection, prognostic stratification, and MRD-informed postoperative management.

Despite these encouraging findings, several limitations warrant consideration. First, although validated in independent cohorts, the sample sizes, especially for certain BTC subtypes and early-stage patients, remain modest. Larger, multi-center prospective studies are needed to confirm the generalizability and robustness of our approach across diverse populations and clinical settings. Second, although the non-cancer group included both healthy individuals and patients with benign biliary diseases, healthy individuals constituted the majority. This imbalance may have led to an overestimation of the model’s discriminatory performance in real-world clinical settings, where distinguishing BTC from benign biliary conditions poses a greater diagnostic challenge. While the external validation cohort partly addressed this limitation, future studies with more balanced control groups are warranted. Third, although the external cohort was collected from a different hospital, sequencing was performed under the same technical conditions, which supports repeatability but does not fully guarantee generalizability to independent laboratory pipelines. Evaluation using truly independent external datasets generated with different pre-analytical and analytical workflows is needed to further confirm the robustness of the model. Fourth, the exact timing of post-surgical plasma collection was not fully standardized across all patients, which may introduce variability in risk score assessment. Future studies with more strictly controlled longitudinal sampling are needed to better define the optimal time points and enhance MRD monitoring accuracy. Finally, the current study did not incorporate complementary biomarkers such as methylation patterns or mutation profiles, which may further enhance detection accuracy when integrated with fragmentomics features. Future multi-omics studies may yield more comprehensive and sensitive tools for BTC early detection and surveillance.

In conclusion, our study demonstrates that integrating multiple cfDNA fragmentomic features via machine learning achieves highly accurate and non-invasive early detection of BTC. The model’s robust performance across diverse patient cohorts, including its ability to distinguish malignant from benign biliary conditions, underscores its clinical potential. Moreover, the prognostic significance of cfDNA-based risk scores before and after surgery highlights its promise for MRD monitoring and personalized patient management. These findings pave the way for future prospective studies and the potential integration of cfDNA fragmentomics into routine BTC screening and surveillance.

## Materials and methods

### Participants and samples

A total of 535 participants were enrolled in this study, comprising 267 patients with biliary tract cancer (BTC) and 268 non-cancer individuals. Participants in the training and internal validation cohorts were recruited at Zhongshan Hospital, Fudan University, with enrollment periods spanning from August 2021 to December 2022 and January 2023 to December 2023, respectively. The external validation cohort included participants from the Third Affiliated Hospital of Sun Yat-sen University, enrolled between January and December 2023.

Exclusion criteria for cancer patients included: (1) prior receipt of cancer therapy; (2) ampullary tumors of unknown origin; (3) sequencing data failing quality control (QC); and (4) withdrawal of informed consent. For non-cancer individuals, exclusion criteria were: (1) a history of any type of cancer; (2) sequencing data failing QC; (3) acute inflammation; (4) loss to follow-up; and (5) withdrawal of informed consent. After applying these criteria, the final cohorts comprised 196 participants in the training set (97 BTC and 99 non-cancer), 132 in the internal validation set (66 BTC and 66 non-cancer), and 167 in the external validation set (82 BTC and 85 non-cancer). In both the model development and validation cohorts, non-cancer participants were stratified into two subgroups: individuals with benign biliary tract diseases and those who were clinically healthy without any known underlying conditions.

All participants underwent blood sampling, and plasma cfDNA was extracted and subjected to low-pass WGS. The study protocol was approved by the Ethics Committee of the participating hospitals (Zhongshan Hospital, Fudan University, Approval No. B2023—299R2; The Third Affiliated Hospital of Sun Yat-Sen University, Approval No. II2024—378—01) and was conducted in accordance with international guidelines for good clinical practice. Written informed consent was obtained from all participants before sample collection.

### Sample processing and whole-genome sequencing

Each participant provided approximately 10 mL of peripheral blood, which was processed for cfDNA isolation within 48 h to preserve sample integrity. Plasma was separated using a two-step centrifugation protocol: an initial centrifugation at 1,600 g for 10 min at 4℃, followed by centrifugation of the supernatant at 16,000 g for 10 min at 4℃. cfDNA was extracted using the GENESEEQMag Cell-Free DNA Isolation Kit (Geneseeq Medical Device and Diagnostic Inc., China) on the Hamilton Microlab STAR automated platform (Hamilton, United States) and quantified with the Qubit dsDNA HS Assay Kit (Thermo Fisher Scientific, United States). Extracted cfDNA samples were stored at −80 °C until further processing. Libraries were constructed using 5—10 ng of cfDNA per sample and reagents from the Sample Collection Kit, automated on the Biomek platform (Beckman Coulter, United States), and quantified using Quantitative PCR reagents (Vazyme, China). WGS was performed with 150 bp paired-end reads on the DNBSEQ-T7 platform, targeting a mean coverage of 5 × for reliable fragmentomic profiling. Only samples meeting predefined QC criteria were included in downstream analyses, including adapter dimer rate ≤ 0.005%, Q30 ≥ 95%, GC content 40—44%, clean base ratio ≥ 96%, mapping ratio ≥ 99%, insert size 150—200 bp, mean depth ≥ 5x, and duplicate rate ≤ 4%. To standardize sequencing depth and mitigate potential confounding, samples with a mean depth > 5 × were downsampled to 5 ×. The training and validation cohorts were sequenced in separate batches, and each batch included both cancer and non-cancer samples, which were randomized and handled using identical reagents and protocols.

### Fragmentomic feature generation

Three cfDNA fragmentomic features were used to construct the diagnostic model: copy number variation (CNV), fragment size distribution (FSD), and promoter fragmentation entropy (PFE).

#### Copy number variation (CNV)

CNV features were derived using ichorCNA following the approach described by Wan et al. [31]. Briefly, the genome was partitioned into 1 Mb bins (2,475 bins in total), and GC/mappability–corrected read counts were obtained for each bin. Bin-level log2 copy ratios were computed relative to the fixed baseline reference provided by ichorCNA, which was generated from 27 healthy-donor cfDNA samples sequenced by 0.1 × ULP-WGS using the same protocol [32]. For each sample, ichorCNA computed the bin-level log2 copy ratio as *l*_*t*_ = log_2_(*r*_*t*_/*h*_*t*_), where *r*_*t*_ is the normalized read depth of the sample at bin *t* and *h*_*t*_ is the corresponding baseline value. Segmentation was performed using a Hidden Markov Model implemented in ichorCNA to infer copy-number states from the bin-level log2 ratios. The same ichorCNA pipeline and baseline reference were applied uniformly to all samples across cohorts.

#### Fragment size distribution (FSD)

The FSD feature captures cfDNA fragment length patterns across the genome. Following the methodology described by Su et al. [33], fragment lengths were binned in 5—bp intervals (100–104 bp, 105–109 bp, …, 215–219 bp), covering the 100–220 bp range. This binning resulted in 24 distinct length intervals per chromosomal arm. Analyses were performed separately for 39 pre-defined chromosome arms (1p, 1q, 2p, 2q, 3p, 3q, 4p, 4q, 5p, 5q, 6p, 6q, 7p, 7q, 8p, 8q, 9p, 9q, 10p, 10q, 11p, 11q, 12p, 12q, 13q, 14q, 15q, 16p, 16q, 17p, 17q, 18p, 18q, 19p, 19q, 20p, 20q, 21q, 22q), selected a priori following the DELFI framework [34]. Chromosome arms with ambiguous chromatin accessibility or unclear boundaries were excluded. This yielded a total of 936 FSD features (24 bins ×39 arms). For each sample, the proportion of fragments within each bin was computed for each arm. To standardize across arms and improve comparability, raw bin-level proportions were converted into z-scores within each sample. This within-sample normalization minimizes cohort dependence and ensures portability of FSD features across datasets. Cancer-associated deviations in fragment size patterns were then assessed by comparing the within-sample z-scores between BTC and non-cancer samples for each bin. This approach captures subtle differences in cfDNA fragmentation patterns that are informative for distinguishing cancer from non-cancer individuals, while avoiding reliance on cohort-level reference distributions.

#### Promoter fragmentation entropy (PFE)

The analysis of the PFE feature followed the method proposed by Esfahani et al. [18]. It is based on the hypothesis that cfDNA fragments originating from active promoters exhibit more random cleavage patterns due to nucleosome displacement or depletion at transcription start sites (TSS) of actively transcribed genes, leading to increased diversity in fragment sizes.

In this study, we first calculated a prior distribution of fragment lengths across the genome, focusing on regions 750 bp to 1 kb upstream and downstream of nucleosome-depleted regions (NDRs). Control entropy values were then calculated by randomly selecting 20 genes from a background control set [35], which consists of 330 genes highly tissue-specific and with very low expression level in the main sources of cfDNA, like blood or common solid-tumor cells, and computing the Shannon entropy of their fragment length distributions. This process was repeated five times to obtain five distinct control entropy values. For each target gene, the fragment length distribution was determined within a 1 kb window flanking its TSS. These distributions were then combined with the prior distribution to form a posterior base distribution, computed as a weighted sum of the prior distribution (weight = 20) and the gene-specific distribution.

Using this posterior base distribution as the parameter for a Dirichlet distribution, 2,000 random samples were drawn, each representing an updated posterior distribution for the gene. Shannon entropy was calculated for each sample, yielding 2,000 posterior entropy values. Each posterior entropy value was then compared to the five control entropy values by calculating the ratio of the posterior to each control. The probability that all five ratios exceeded 1 + k was estimated, where k follows a Gamma distribution, k ~ Γ (s = 0.5, r = 1). The mean value of these probabilities across the 2,000 samples was defined as the PFE score for the gene. This comprehensive approach allows the PFE score to sensitively reflect cfDNA fragmentation patterns, offering insights into promoter-level transcriptional activity. To account for inter-sample variability and ensure comparability across samples, gene-level PFE scores were normalized within each sample, consistent with the normalization strategy described by Esfahani et al. [18]. This within-sample normalization preserves relative promoter entropy patterns while mitigating global entropy shifts attributable to technical variation.

Feature selection was then performed exclusively in the training cohort based on these normalized PFE values. Candidate PFE features were identified using Wilcoxon rank-sum tests, selecting genes with *p* < 0.01 and a positive mean difference between cancer and non-cancer samples. This procedure yielded a fixed set of 1,203 PFE features, which were defined a priori and consistently applied in downstream analyses (Additional file 1). GO and KEGG enrichment analyses were performed independently on this gene set using clusterProfiler with Benjamini–Hochberg adjustment for multiple testing, to illustrate the potential biological functions of the PFE-selected genes without influencing feature selection.

### PFE downsampling analysis

To evaluate the robustness of PFE features under low-pass WGS, a depth-stability analysis was performed in the validation cohorts. Within each cohort, samples were first stratified by status (cancer vs non-cancer). For each stratum, BAM files from individual samples were merged to construct composite reference samples with a mean sequencing depth of at least 50x. Because higher-depth merging would reduce sample numbers and gene expression-PFE correlations plateau around 50 × [18], we selected 50 × as a practical reference depth. In total, 36 composite reference samples were generated (internal validation cohort: 7 cancer and 8 non-cancer; external validation cohort: 10 cancer and 11 non-cancer).

For each composite reference sample, random downsampling was performed to generate datasets at five target sequencing depths: 40x, 30x, 20x, 10x, and 5x, using the original 50 × composite as the reference depth. PFE features were recalculated independently at each depth using the same computational pipeline described above.

To assess depth stability, PFE feature profiles derived at each lower depth were compared with those obtained at the corresponding 50 × reference depth from the same composite sample. Pearson correlation coefficients were calculated to quantify concordance across sequencing depths, thereby evaluating the robustness of entropy-based promoter fragmentation signals under reduced sequencing coverage.

### Model construction

The H2O AutoML framework (version 3.36.0.3) was used for model construction [36]. Fragmentomic features (CNV, FSD, and PFE) are heterogeneous in scale and distribution and may have complex nonlinear relationships with disease status. To balance interpretability and predictive performance, we evaluated models spanning linear, tree-based ensemble, and deep learning approaches, including generalized linear model (GLM), gradient boosting machine (GBM), random forest (RF), deep learning (DL), and XGBoost (XGB). GLM was included as a baseline linear model for interpretability and robustness. Tree-based ensemble methods (GBM, RF, and XGB) can capture nonlinear relationships and feature interactions, are robust to feature scaling, and handle correlated or sparse variables typical of fragmentomic data. DL models were assessed to explore whether hierarchical nonlinear representations could further improve performance. Shallow fully connected networks (1–3 hidden layers, 50–200 units per layer) were automatically configured by AutoML with randomized activation functions (Rectifier or Tanh, with or without dropout), input/hidden dropout, and L1/L2 regularization; no manual architecture tuning was performed.

For each feature matrix, H2O AutoML performed a randomized hyperparameter search, generating approximately 200 candidate models. Algorithm-specific hyperparameters were explored using H2O AutoML’s default randomized search strategy. For GLM, α ∈ [0,1] with λ automatically generated; for GBM and RF, standard tree-based hyperparameters such as ntrees, max_depth, min_rows, sample_rate, and column sampling were explored. Deep learning and XGBoost models were automatically configured by AutoML within algorithm-specific default ranges, without manual tuning. All candidate models were trained using fivefold cross-validation within the training cohort, and out-of-fold predictions (OOF) were saved for subsequent model ranking and ensemble construction, ensuring that validation cohorts remained fully independent and preventing information leakage.

Base models were ranked according to the AUC computed from their aggregated OOF predictions across all training samples, and the top 10 models with the highest AUC were selected for ensemble construction. For each sample, the predictions of these top models were averaged to obtain a per-feature ensemble score. The top models may include StackedEnsemble variants, which combine predictions from multiple base learners across algorithm families. Their inclusion reflects performance-driven selection rather than exclusion of any particular algorithm. Thus, the fundamental modeling unit in our framework is the performance-ranked base learner, rather than a pre-specified algorithm category. The 30 selected base models (10 per feature matrix), along with their algorithm class and Train_AUC, are listed in Table S10. Finally, the BTC risk score was computed as the equal-weight average of the three per-feature ensemble scores, integrating CNV, FSD, and PFE information. Validation cohorts were withheld during all training, hyperparameter search, model selection, and ensemble construction steps, ensuring that performance metrics represent independent evaluation.

### Statistical analysis

All statistical analyses were conducted using R software (v4.3.2). Categorical variables were compared using the Chi-square test or Fisher’s Exact test, as appropriate. For pairwise comparisons of risk scores between two groups, we used two-sided Wilcoxon rank-sum tests. Effect size was summarized by the Wilcoxon *r* statistic, with 95% confidence intervals (CIs) obtained by nonparametric bootstrap resampling within groups (2,000 replicates; percentile method). For multiple comparisons, p-values were adjusted using the Benjamini–Hochberg procedure, and the resulting q-values are reported. The “epiR” package was employed to calculate diagnostic performance metrics, including true positives (TP), true negatives (TN), false positives (FP), and false negatives (FN), from which sensitivity, specificity, and accuracy were derived, along with their corresponding 95% CIs. Receiver operating characteristic (ROC) curves were generated, and area under the curve (AUC) values were computed using the “pROC” package. DeLong’s test was used to compare AUCs between different models or subgroups when applicable. Disease-free survival (DFS) was defined as the date from surgery to disease recurrence or death by any cause. Overall survival (OS) was defined as the time from diagnosis until death from any cause. Event-time distributions were visualized using Kaplan–Meier survival curves, with statistical differences assessed using the log-rank test. Cox proportional hazard models were fitted to estimate hazard ratios (HRs) with 95% CIs. The proportional hazards assumption for Cox regression models was assessed using Schoenfeld residuals. Both global and covariate-specific tests were performed using the cox.zph function in the R survival package, and a p-value > 0.05 was considered evidence of no violation of the proportional hazards assumption. Model calibration was assessed using calibration curves, calibration intercept, calibration slope, and Brier score to evaluate the agreement between predicted probabilities and observed outcomes. Unless otherwise specified, all reported *P*-values were two-tailed, with statistical significance defined as *P* < 0.05.

## Supplementary Information

Supplementary Material 1

Supplementary Material 2

## Data Availability

The datasets generated and/or analyzed during the current study are available in the Genome Sequence Archive (GSA) for Human under accession number HRA013047. Access to these data can be obtained from the corresponding author (liuhbfdu@163.com) upon reasonable request.
