# Supplementary material for: Noninvasive detection and prognostic stratification of biliary tract cancer using cell-free DNA fragmentomics: a model development and validation study
Source: Mol Biomed. 2026 May 21;7:72. doi: 10.1186/s43556-026-00468-7 (PMC13194824; doi:10.1186/s43556-026-00468-7)
Supplement: Supplementary file 2 — Supplementary Material 2 [file 43556_2026_468_MOESM2_ESM.docx]

**Supplementary Materials**

**Manuscript Title: Noninvasive detection and prognostic stratification of biliary tract cancer using cell-free DNA fragmentomics: a model development and validation study**

Jiwen Wang^1, 2, 3, 4, 5, 6*^, Xiaojian Ni^1, 2, 3, 4, 5*^, Yuxuan Zheng^1, 2, 3, 4, 5*^, Song Wang^7*^, Hua Bao^7^, Kun Fan^6^, Sheng Shen^1, 2, 3, 4, 5^, Dongqin Zhu^7^, Qingxin Xie^7^, Hairong Bao^7^, Ruowei Yang^7^, Chunyan Wang^6^, Bohao Zheng^1, 2, 3, 4, 5^, Shuang Chang^7^, Xiuxiu Xu^7^, Xiaoling Ni^1, 2, 3, 4, 5^, Tao Suo^1, 2, 3, 4, 5^, Xue Wu^7^, Han Liu^1, 2, 3, 4, 5#^, Xiaokun Ma^8#^, Houbao Liu^1, 2, 3, 4, 5, 6#^.

1 Department of Biliary Surgery, Zhongshan Hospital, Fudan University, Shanghai 200032, China

2 Biliary Tract Disease Center of Zhongshan Hospital, Fudan University, Shanghai 200032, China

3 Cancer Center, Zhongshan Hospital, Fudan University, Shanghai 200032, China

4 Biliary Tract Disease Institute, Fudan University, Shanghai 200032, China

5 Shanghai Engineering Research Center of Biliary Tract Minimal Invasive Surgery and Materials, Shanghai 200032, China

6 Department of General Surgery, Zhongshan-Xuhui Hospital, Fudan University, Shanghai 200030, China

7 Geneseeq Research Institute, Nanjing Geneseeq Technology Inc., Nanjing 210032, China

8 Department of Medical Oncology, The Third Affiliated Hospital of Sun Yat-Sen University, Guangzhou, 510630, China

* These authors contributed equally to this work.

# Corresponding Authors

**Houbao Liu**, MD, PhD, Department of Biliary Surgery, Zhongshan Hospital, Fudan University, 180 Fenglin Road, Shanghai, 200032, China. E-mail: liuhbfdu@163.com

**Xiaokun Ma**, MD, Department of Medical Oncology, The Third Affiliated Hospital of Sun Yat-Sen University, 510630, China. E-mail: maxiaok@mail.sysu.edu.cn

**Han Liu**, MD, PhD, Department of Biliary Surgery, Zhongshan Hospital, Fudan University, 180 Fenglin Road, Shanghai, 200032, China. E-mail: liu.han@zs-hospital.sh.cn

## **Supplementary Tables**

| **Table S1. Short cfDNA fragment enrichment across clinical stages in the training cohort** | | | |
| --- | --- | --- | --- |
| Fragment size | P-value | Test | Alternative |
| 100-104 | < 1e-16 | Jonckheere-Terpstra test | Increasing |
| 105-109 | < 1e-16 | Jonckheere-Terpstra test | Increasing |
| 110-114 | < 1e-16 | Jonckheere-Terpstra test | Increasing |
| 115-119 | < 1e-16 | Jonckheere-Terpstra test | Increasing |
| 120-124 | < 1e-16 | Jonckheere-Terpstra test | Increasing |
| 125-129 | < 1e-16 | Jonckheere-Terpstra test | Increasing |
| 130-134 | < 1e-16 | Jonckheere-Terpstra test | Increasing |
| 135-139 | < 1e-16 | Jonckheere-Terpstra test | Increasing |
| 140-144 | < 1e-16 | Jonckheere-Terpstra test | Increasing |
| 145-149 | < 1e-16 | Jonckheere-Terpstra test | Increasing |

| **Table S2. Subgroup comparisons of risk scores with effect size estimates** | | | | | | | | | |
| --- | --- | --- | --- | --- | --- | --- | --- | --- | --- |
| Subgroup | Dataset | Group1 | Group2 | n1 | n2 | p-value | q-value | q_signif | Effect size (95% CI) |
| GroupLevel1 | Train | Cancer | Noncancer | 97 | 99 | 2.0171E-31 | 2.0171E-31 | *** | 0.833 (0.807, 0.853) |
|  | Internal | Cancer | Noncancer | 66 | 66 | 1.0241E-19 | 1.0241E-19 | *** | 0.791 (0.728, 0.835) |
|  | External | Cancer | Noncancer | 82 | 85 | 1.6354E-22 | 1.6354E-22 | *** | 0.756 (0.687, 0.808) |
| GroupLevel2 | Train | Cancer | Disease | 97 | 25 | 2.4869E-13 | 3.7304E-13 | *** | 0.663 (0.627, 0.687) |
|  | Train | Cancer | Healthy | 97 | 74 | 2.3413E-27 | 7.0239E-27 | *** | 0.829 (0.800, 0.849) |
|  | Train | Disease | Healthy | 25 | 74 | 0.89428125 | 0.89428125 | ns | 0.014 (-0.187, 0.215) |
|  | Internal | Cancer | Disease | 66 | 16 | 4.2084E-08 | 6.3126E-08 | *** | 0.606 (0.530, 0.662) |
|  | Internal | Cancer | Healthy | 66 | 50 | 1.6554E-17 | 4.9663E-17 | *** | 0.791 (0.732, 0.832) |
|  | Internal | Disease | Healthy | 16 | 50 | 0.07621664 | 0.07621664 | ns | 0.219 (-0.004, 0.415) |
|  | External | Cancer | Disease | 82 | 56 | 1.7503E-17 | 5.2508E-17 | *** | 0.725 (0.646, 0.789) |
|  | External | Cancer | Healthy | 82 | 29 | 3.0312E-13 | 4.5468E-13 | *** | 0.693 (0.632, 0.736) |
|  | External | Disease | Healthy | 56 | 29 | 0.04576647 | 0.04576647 | * | 0.217 (0.004, 0.421) |
| Stage | Train | II | III | 65 | 18 | 0.04609683 | 0.13829049 | ns | 0.220 (-0.009, 0.425) |
|  | Train | II | I | 65 | 14 | 0.22502737 | 0.33754106 | ns | 0.137 (-0.090, 0.345) |
|  | Train | III | I | 18 | 14 | 0.39271486 | 0.39271486 | ns | -0.154 (-0.498, 0.202) |
|  | Internal | II | I | 46 | 10 | 0.66098103 | 0.66098103 | ns | 0.060 (-0.240, 0.363) |
|  | Internal | II | III | 46 | 10 | 0.22677362 | 0.66098103 | ns | 0.163 (-0.077, 0.392) |
|  | Internal | I | III | 10 | 10 | 0.57075039 | 0.66098103 | ns | 0.135 (-0.339, 0.577) |
|  | External | III | II | 19 | 47 | 0.00422649 | 0.00422649 | ** | 0.353 (0.154, 0.529) |
|  | External | III | I | 19 | 16 | 3.8502E-06 | 1.1551E-05 | *** | 0.784 (0.650, 0.852) |
|  | External | II | I | 47 | 16 | 0.00086285 | 0.00129428 | ** | 0.421 (0.227, 0.587) |
| Histology | Train | ADC | ASC | 94 | 3 | 0.56661691 | 0.56661691 | ns | -0.059 (-0.248, 0.254) |
|  | Internal | ADC | ASC | 64 | 2 | 0.08196321 | 0.08196321 | ns | 0.216 (0.166, 0.263) |
|  | External | ADC | ASC | 75 | 7 | 0.01111642 | 0.01111642 | * | -0.281 (-0.417, -0.135) |
| Subsite | Train | GBC | dCCA | 32 | 46 | 0.00011569 | 0.00069411 | *** | -0.437 (-0.604, -0.243) |
|  | Train | GBC | pCCA | 32 | 16 | 0.07467731 | 0.22403194 | ns | -0.259 (-0.518, 0.010) |
|  | Train | GBC | iCCA | 32 | 3 | 0.61646452 | 0.73975742 | ns | -0.090 (-0.389, 0.299) |
|  | Train | dCCA | pCCA | 46 | 16 | 0.18982068 | 0.37964137 | ns | 0.168 (-0.100, 0.409) |
|  | Train | dCCA | iCCA | 46 | 3 | 0.26910483 | 0.40365724 | ns | 0.161 (-0.030, 0.393) |
|  | Train | pCCA | iCCA | 16 | 3 | 0.95542012 | 0.95542012 | ns | 0.026 (-0.389, 0.541) |
|  | Internal | dCCA | GBC | 22 | 23 | 0.02531776 | 0.03797665 | * | 0.335 (0.061, 0.593) |
|  | Internal | dCCA | pCCA | 22 | 21 | 0.21083652 | 0.21083652 | ns | -0.193 (-0.486, 0.104) |
|  | Internal | GBC | pCCA | 23 | 21 | 0.00414876 | 0.01244629 | * | -0.434 (-0.665, -0.161) |
|  | External | iCCA | dCCA | 22 | 30 | 0.43118117 | 0.64677176 | ns | 0.110 (-0.177, 0.403) |
|  | External | iCCA | GBC | 22 | 17 | 0.11597971 | 0.59085082 | ns | 0.254 (-0.059, 0.545) |
|  | External | iCCA | pCCA | 22 | 13 | 0.95915886 | 0.95915886 | ns | 0.012 (-0.318, 0.335) |
|  | External | dCCA | GBC | 30 | 17 | 0.20693522 | 0.59085082 | ns | 0.186 (-0.091, 0.452) |
|  | External | dCCA | pCCA | 30 | 13 | 0.76104456 | 0.91325347 | ns | -0.048 (-0.379, 0.279) |
|  | External | GBC | pCCA | 17 | 13 | 0.29542541 | 0.59085082 | ns | -0.195 (-0.540, 0.149) |
| Stage-stratified  BTC vs noncancer (healthy/disease) | Train | Stage I BTC | Disease | 14 | 25 | 5.1402E-07 | 7.7102E-07 | *** | 0.806 (0.765, 0.822) |
|  | Train | Stage I BTC | Healthy | 14 | 74 | 6.1562E-09 | 1.8468E-08 | *** | 0.620 (0.597, 0.630) |
|  | Internal | Stage I BTC | Disease | 10 | 16 | 0.00045685 | 0.00068528 | *** | 0.693 (0.466, 0.829) |
|  | Internal | Stage I BTC | Healthy | 10 | 50 | 7.0321E-06 | 2.1096E-05 | *** | 0.581 (0.494, 0.640) |
|  | External | Stage I BTC | Disease | 16 | 56 | 0.00027605 | 0.00041407 | *** | 0.429 (0.220, 0.600) |
|  | External | Stage I BTC | Healthy | 16 | 29 | 3.1643E-05 | 9.4929E-05 | *** | 0.622 (0.428, 0.771) |
|  | Train | Stage II BTC | Disease | 65 | 25 | 1.2608E-12 | 1.8911E-12 | *** | 0.749 (0.718, 0.769) |
|  | Train | Stage II BTC | Healthy | 65 | 74 | 2.5815E-23 | 7.7444E-23 | *** | 0.844 (0.823, 0.858) |
|  | Internal | Stage II BTC | Disease | 46 | 16 | 6.7663E-08 | 1.015E-07 | *** | 0.686 (0.599, 0.752) |
|  | Internal | Stage II BTC | Healthy | 46 | 50 | 2.0373E-15 | 6.1119E-15 | *** | 0.811 (0.752, 0.851) |
|  | External | Stage II BTC | Disease | 47 | 56 | 1.2554E-14 | 3.7661E-14 | *** | 0.760 (0.673, 0.823) |
|  | External | Stage II BTC | Healthy | 47 | 29 | 1.041E-11 | 1.5615E-11 | *** | 0.781 (0.717, 0.824) |
|  | Train | Stage III BTC | Disease | 18 | 25 | 1.9013E-06 | 2.852E-06 | *** | 0.728 (0.594, 0.823) |
|  | Train | Stage III BTC | Healthy | 18 | 74 | 2.1108E-09 | 6.3323E-09 | *** | 0.625 (0.548, 0.675) |
|  | Internal | Stage III BTC | Disease | 10 | 16 | 0.00055613 | 0.0008342 | *** | 0.682 (0.425, 0.829) |
|  | Internal | Stage III BTC | Healthy | 10 | 50 | 1.6018E-05 | 4.8055E-05 | *** | 0.558 (0.428, 0.640) |
|  | External | Stage III BTC | Disease | 19 | 56 | 1.2097E-10 | 3.6292E-10 | *** | 0.744 (0.732, 0.749) |
|  | External | Stage III BTC | Healthy | 19 | 29 | 7.6255E-09 | 1.1438E-08 | *** | 0.835 (0.821, 0.839) |
|  | Train | Disease | Healthy | 25 | 74 | 0.89428125 | 0.89428125 | ns | 0.014 (-0.186, 0.223) |
|  | Internal | Disease | Healthy | 16 | 50 | 0.076216637 | 0.076216637 | ns | 0.219 (0.007, 0.415) |
|  | External | Disease | Healthy | 56 | 29 | 0.045766466 | 0.045766466 | * | 0.217 (0.012, 0.426) |
| q_signif: statistical significance based on FDR-adjusted p-values (q-values), with thresholds as follows: ns, q > 0.05; *, q < 0.05; **, q < 0.01; ***, q < 0.001.  Effect size direction: positive = group 1 > group2, negative = group2 > group 1. | | | | | | | | | |

| **Table S3. Performance metrics across datasets** | | | | | | | |
| --- | --- | --- | --- | --- | --- | --- | --- |
| **Cutoff = 0.465 corresponds to 95% specificity in the training set** | | | | | | | |
|  | | **Training set** | | **Internal validation set** | | **External validation set** | |
|  |  | **Actual** | | **Actual** | | **Actual** | |
|  |  | **BTC** | **Non-cancer** | **BTC** | **Non-cancer** | **BTC** | **Non-cancer** |
| **Predict** | **BTC** | 89 | 5 | 52 | 6 | 65 | 5 |
|  | **Non-cancer** | 8 | 94 | 14 | 60 | 17 | 80 |
| **Sensitivity (95% CI)** | | 0.918 (0.844-0.964) | | 0.788 (0.67-0.879) | | 0.793 (0.689-0.874) | |
| **Specificity (95% CI)** | | 0.949 (0.886-0.983) | | 0.909 (0.813-0.966) | | 0.941 (0.868-0.981) | |
| **PPV (95% CI)** | | 0.947 (0.880-0.983) | | 0.897 (0.788-0.961) | | 0.929 (0.841-0.976) | |
| **NPV (95% CI)** | | 0.922 (0.851-0.966) | | 0.811 (0.703-0.893) | | 0.825 (0.734-0.894) | |
| **Accuracy (95% CI)** | | 0.934 (0.889-0.964) | | 0.848 (0.776-0.905) | | 0.868 (0.807-0.916) | |

| **Table S4. Scenario analysis of PPV and NPV for the model under varying disease prevalence** | | | | | |
| --- | --- | --- | --- | --- | --- |
|  | Sensitivity | Specificity | Prevalence (%) | PPV | NPV |
| Train | 0.949 (0.884-0.983) | 0.889 (0.810-0.943) | 0.1 | 0.008 | 1 |
|  |  |  | 1 | 0.080 | 0.999 |
|  |  |  | 5 | 0.310 | 0.997 |
|  |  |  | 10 | 0.487 | 0.994 |
|  |  |  | 20 | 0.681 | 0.986 |
| Internal validation | 0.833 (0.721-0.914) | 0.909 (0.813-0.966) | 0.1 | 0.009 | 1 |
|  |  |  | 1 | 0.085 | 0.998 |
|  |  |  | 5 | 0.328 | 0.99 |
|  |  |  | 10 | 0.505 | 0.98 |
|  |  |  | 20 | 0.696 | 0.956 |
| External validation | 0.842 (0.744-0.913) | 0.906 (0.823-0.959) | 0.1 | 0.009 | 1 |
|  |  |  | 1 | 0.083 | 0.998 |
|  |  |  | 5 | 0.321 | 0.991 |
|  |  |  | 10 | 0.497 | 0.981 |
|  |  |  | 20 | 0.693 | 0.958 |
| Abbreviations: PPV, positive predictive value; NPV, negative predictive value | | | | | |

| **Table S5. Stage I BTC model specificity versus disease or healthy controls** | | | |
| --- | --- | --- | --- |
| Dataset | Subgroup | Specificity | 95% confidence interval |
| Train | Stage I vs Disease | 0.800 | 0.64 - 0.96 |
| Train | Stage I vs Healthy | 0.919 | 0.851 - 0.973 |
| Internal valid | Stage I vs Disease | 0.875 | 0.688 – 1.000 |
| Internal valid | Stage I vs Healthy | 0.920 | 0.84 - 0.98 |
| External valid | Stage I vs Disease | 0.875 | 0.786 - 0.946 |
| External valid | Stage I vs Healthy | 0.966 | 0.897 – 1.000 |

| **Table S6. Clinical characteristics of external postoperative BTC cohort (N=78)** | |
| --- | --- |
| Age | 63.5 [39.0, 74.0] |
| Sex |  |
| Male | 44 (56.4%) |
| Female | 34 (43.6%) |
| Clinical stage | |
| I | 15 (19.2%) |
| II | 46 (59.0%) |
| III | 17 (21.8%) |
| Anatomical type |  |
| iCCA | 19 (24.4%) |
| pCCA | 13 (16.7%) |
| dCCA | 29 (37.2%) |
| GBC | 17 (21.8%) |
| Histology |  |
| Adenocarcinoma | 71 (91.0%) |
| Adenosquamous carcinoma | 7 (9.0%) |
| Differentiation | |
| Poor | 45 (57.7%) |
| Moderate | 28 (35.9%) |
| Well | 0 (0%) |
| Unknown | 5 (6.4%) |
| WBC (10^9^/L) |  |
| Median (range) | 6.18 [3.31, 11.1] |
| > 10 | 3 (3.8%) |
| ≤ 10 | 75 (96.2%) |
| DB (µmol/L) |  |
| Median (range) | 24.5 [1.40, 93.0] |
| > 6.8 | 53 (67.9%) |
| ≤ 6.8 | 25 (32.1%) |
| Max tumor diameter (cm) |  |
| Median (range) | 2.50 [0.700, 11.5] |
| Postoperative complications | |
| No | 44 (56.4%) |
| Yes | 34 (43.6%) |
| Sampling time | |
| 1 week | 75 (96.2%) |
| 1 month | 2 (2.6%) |
| 3 months | 1 (1.3%) |

| **Table S7. Univariate Cox analysis for disease-free survival** | | | | |
| --- | --- | --- | --- | --- |
| Feature | Comparison | HR | 95% CI | P-value |
| Sex | Male vs Female | 0.574 | 0.282 - 1.167 | 0.125 |
| Age | Continuous | 0.974 | 0.925 - 1.024 | 0.303 |
| Age_group | ≥ 60 years vs < 60 years | 0.921 | 0.433 - 1.96 | 0.832 |
| Stage | II vs I | 2.435 | 0.708 - 8.378 | 0.158 |
|  | III vs I | 7.100 | 1.985 - 25.395 | 0.003** |
| Histology | ADC vs ASC | 0.268 | 0.115 - 0.624 | 0.002** |
| Subsite | iCCA vs GBC | 1.245 | 0.471 - 3.288 | 0.658 |
|  | pCCA vs GBC | 0.295 | 0.06 - 1.435 | 0.13 |
|  | dCCA vs GBC | 1.011 | 0.394 - 2.591 | 0.982 |
| Subsite_group | GBC vs Non-GBC | 1.100 | 0.469 - 2.577 | 0.827 |
| Differentiation | Moderate vs Poor | 0.348 | 0.14 - 0.864 | 0.023* |
|  | Unknown vs Poor | 1.655 | 0.566 - 4.843 | 0.357 |
| WBC (10^9^/L) | Continuous | 1.065 | 0.876 - 1.296 | 0.527 |
|  | >10 vs ≤10 | 0.888 | 0.121 - 6.524 | 0.907 |
| DB (µmol/L) | Continuous | 0.993 | 0.978 - 1.009 | 0.394 |
|  | > 6.8 vs ≤ 6.8 | 1.088 | 0.512 - 2.312 | 0.827 |
| Sampling time | One-month vs One-week | 0 | 0 - Inf | 0.998 |
|  | Three-month vs One-week | 0 | 0 - Inf | 0.998 |
|  | Others vs One-week | 0 | 0 - Inf | 0.997 |
| Postoperative  complications | Yes vs No | 1.450 | 0.7 - 3.005 | 0.317 |
| Max diameter (cm) | Continuous | 1.154 | 1.018 - 1.308 | 0.025* |
| Postoperative risk score | High-risk vs Low-risk | 4.772 | 2.038 - 11.173 | < 0.001*** |
| Baseline risk score | High-risk vs Low-risk | 4.060 | 0.967 - 17.056 | 0.056 |
| Postoperative risk score | Continuous | 11.226 | 2.314 - 54.450 | 0.003** |
| Baseline risk score | Continuous | 40.379 | 5.743 - 283.899 | < 0.001*** |
| Abbreviations: HR, hazard ratio; CI, confidence interval; WBC, white blood cell; DB, direct bilirubin  *, p < 0.05; **, p < 0.01; ***, p < 0.001 | | | | |

| **Table S8. Variance inflation factors (VIFs) for covariates across Cox regression models** | | |
| --- | --- | --- |
| Model | Term | VIF |
| Primary model | Stage (III vs I-II) | 1.653072 |
|  | Max diameter | 2.410857 |
|  | WBC | 1.199266 |
|  | Log(DB) | 1.28028 |
|  | Age | 1.226183 |
|  | Histology (ADC vs ASC) | 1.608209 |
|  | Postoperative risk score (High-risk vs Low-risk) | 1.08009 |
| Complication-adjusted model | Stage (III vs I-II) | 1.694313 |
|  | Max diameter | 2.480046 |
|  | WBC | 1.228833 |
|  | Log(DB) | 1.308451 |
|  | Age | 1.230563 |
|  | Histology (ADC vs ASC) | 1.639901 |
|  | Postoperative risk score (High-risk vs Low-risk) | 1.088043 |
|  | Postoperative complications (Yes vs No) | 1.216759 |
| Subsite-stratified model | Stage (III vs I-II) | 1.501453 |
|  | Max diameter | 2.105401 |
|  | WBC | 1.192366 |
|  | Log(DB) | 1.249052 |
|  | Age | 1.174817 |
|  | Histology (ADC vs ASC) | 1.563269 |
|  | Postoperative risk score (High-risk vs Low-risk) | 1.09696 |
| Alternative staging model | Stage (II vs I) | 3.57013 |
|  | Stage (III vs I) | 3.501023 |
|  | Max diameter | 2.919029 |
|  | WBC | 1.209278 |
|  | Log(DB) | 1.280873 |
|  | Age | 1.245498 |
|  | Histology (ADC vs ASC) | 1.778515 |
|  | Postoperative risk score (High-risk vs Low-risk) | 1.07578 |
| Categorized WBC/DB model | Stage (III vs I-II) | 1.687998 |
|  | Max diameter | 2.328393 |
|  | WBC_group (>10 vs ≤10) | 1.087469 |
|  | DB_group (>6.8 vs ≤6.8) | 1.488202 |
|  | Age | 1.198882 |
|  | Histology (ADC vs ASC) | 1.721007 |
|  | Postoperative risk score (High-risk vs Low-risk) | 1.095913 |
| Unified sampling time model | Stage (III vs I-II) | 1.624483 |
|  | Max diameter | 2.413577 |
|  | WBC | 1.179733 |
|  | Log(DB) | 1.273588 |
|  | Age | 1.207532 |
|  | Histology (ADC vs ASC) | 1.614934 |
|  | Postoperative risk score (High-risk vs Low-risk) | 1.069378 |
| Abbreviations: WBC, white blood cell count; DB, direct bilirubin; ADC, adenocarcinoma; ASC, Adenosquamous carcinoma | | |

| **Table S9. Schoenfeld residual tests for proportional hazards across multivariable Cox models** | | | | |
| --- | --- | --- | --- | --- |
| Model | Covariate | chisq | df | p |
| Primary model | Stage (two levels) | 2.34206044 | 1 | 0.126 |
|  | Max diameter | 0.635299488 | 1 | 0.425 |
|  | WBC | 0.113144793 | 1 | 0.737 |
|  | log(DB) | 2.900405266 | 1 | 0.089 |
|  | Age | 0.086770435 | 1 | 0.768 |
|  | Histology | 0.025136522 | 1 | 0.874 |
|  | Postoperative risk score | 0.610498504 | 1 | 0.435 |
|  | GLOBAL | 9.161920528 | 7 | 0.241 |
| Complications-adjusted model | Stage (two levels) | 2.309715437 | 1 | 0.129 |
|  | Max diameter | 0.583070138 | 1 | 0.445 |
|  | WBC | 0.130387156 | 1 | 0.718 |
|  | log(DB) | 2.851973799 | 1 | 0.091 |
|  | Age | 0.070737358 | 1 | 0.790 |
|  | Histology | 0.045738083 | 1 | 0.831 |
|  | Postoperative risk score | 0.649366662 | 1 | 0.420 |
|  | Postoperative complications | 2.335622137 | 1 | 0.126 |
|  | GLOBAL | 10.12173405 | 8 | 0.257 |
| Subsite-stratified model | Stage (two levels) | 0.440474776 | 1 | 0.507 |
|  | Max diameter | 0.052049876 | 1 | 0.820 |
|  | WBC | 0.193574264 | 1 | 0.660 |
|  | log(DB) | 1.496112307 | 1 | 0.221 |
|  | Age | 0.069029082 | 1 | 0.793 |
|  | Histology | 0.180215825 | 1 | 0.671 |
|  | Postoperative risk score | 0.632127477 | 1 | 0.427 |
|  | GLOBAL | 4.236527412 | 7 | 0.752 |
| Alternative staging model | Stage (three levels) | 4.059467728 | 2 | 0.131 |
|  | Max diameter | 0.582455603 | 1 | 0.445 |
|  | WBC | 0.060402098 | 1 | 0.806 |
|  | log(DB) | 2.867422372 | 1 | 0.090 |
|  | Age | 0.067189746 | 1 | 0.795 |
|  | Histology | 0.006474766 | 1 | 0.936 |
|  | Postoperative risk score | 0.64764311 | 1 | 0.421 |
|  | GLOBAL | 11.5085591 | 8 | 0.175 |
| Categorized WBC/DB model | Stage (two levels) | 2.407486572 | 1 | 0.121 |
|  | Max diameter | 0.644862711 | 1 | 0.422 |
|  | WBC_group | 0.594924531 | 1 | 0.441 |
|  | DB_group | 0.559409219 | 1 | 0.454 |
|  | Age | 0.030945807 | 1 | 0.860 |
|  | Histology | 0.003908542 | 1 | 0.950 |
|  | Postoperative risk score | 0.799672851 | 1 | 0.371 |
|  | GLOBAL | 7.569022149 | 7 | 0.372 |
| Unified sampling time model | Stage (two levels) | 2.562459171 | 1 | 0.109 |
|  | Max diameter | 0.722842345 | 1 | 0.395 |
|  | WBC | 0.201591056 | 1 | 0.653 |
|  | log(DB) | 2.697177702 | 1 | 0.101 |
|  | Age | 0.0645025 | 1 | 0.800 |
|  | Histology | 0.019267833 | 1 | 0.890 |
|  | Postoperative risk score | 0.756246213 | 1 | 0.385 |
|  | GLOBAL | 9.092916352 | 7 | 0.246 |
| Abbreviations: WBC, white blood cell count; DB, direct bilirubin; df, degrees of freedom | | | | |

| **Table S10. Top 10 base models per feature matrix used to construct the BTC risk score** | | |
| --- | --- | --- |
| Feature | Algorithm | Train_AUC |
| CNV | StackedEnsemble_BestOfFamily_6 | 0.93908154 |
| CNV | StackedEnsemble_BestOfFamily_5 | 0.93574925 |
| CNV | StackedEnsemble_Best1000_1 | 0.93283349 |
| CNV | StackedEnsemble_BestOfFamily_8 | 0.92913673 |
| CNV | StackedEnsemble_BestOfFamily_4 | 0.92627304 |
| CNV | StackedEnsemble_BestOfFamily_7 | 0.91419348 |
| CNV | GLM_1 | 0.91304801 |
| CNV | GBM_grid_1_model_13 | 0.91096532 |
| CNV | GBM_grid_1_model_36 | 0.90992398 |
| CNV | StackedEnsemble_BestOfFamily_3 | 0.90940331 |
| PFE | XGBoost_lr_search_selection_select_grid_model_4 | 0.94585026 |
| PFE | GBM_grid_1_model_35 | 0.94106009 |
| PFE | GBM_grid_1_model_25 | 0.93262522 |
| PFE | GBM_grid_1_model_26 | 0.93158388 |
| PFE | GBM_grid_1_model_37 | 0.92845986 |
| PFE | GBM_grid_1_model_4 | 0.92044153 |
| PFE | GBM_grid_1_model_3 | 0.91950432 |
| PFE | GBM_grid_1_model_7 | 0.91908779 |
| PFE | GBM_grid_1_model_16 | 0.91773404 |
| PFE | GBM_grid_1_model_20 | 0.91742164 |
| FSD | StackedEnsemble_BestOfFamily_4 | 0.91841091 |
| FSD | StackedEnsemble_BestOfFamily_7 | 0.90992398 |
| FSD | StackedEnsemble_BestOfFamily_6 | 0.90898678 |
| FSD | StackedEnsemble_Best1000_1 | 0.90169739 |
| FSD | GLM_1 | 0.89826096 |
| FSD | StackedEnsemble_BestOfFamily_1 | 0.89826096 |
| FSD | StackedEnsemble_BestOfFamily_2 | 0.89492867 |
| FSD | StackedEnsemble_BestOfFamily_3 | 0.88597313 |
| FSD | StackedEnsemble_BestOfFamily_5 | 0.88446319 |
| FSD | XGBoost_grid_1_model_66 | 0.8248464 |

## **Supplementary Figures**


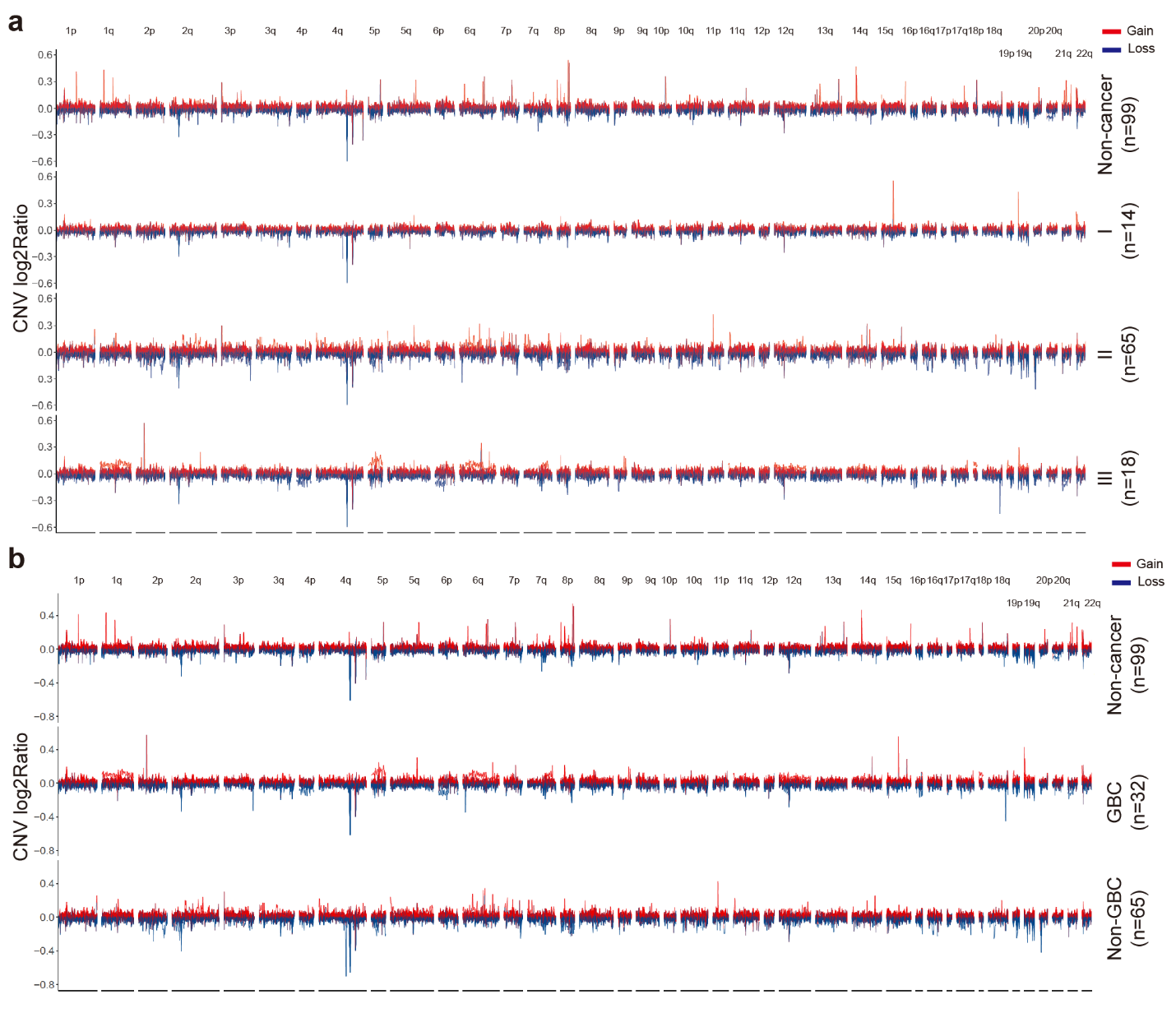


### **Figure S1. Copy number variation profiles**

(**a**) Genome-wide copy number variation (CNV) profiles for non-cancer controls and biliary tract cancer (BTC) patients by clinical stage in the training cohort. Each trace represents the log2 ratio of copy number changes across autosomes, with red indicating copy number gain (log2 ratio > 0), and blue indicating copy number loss (log2 ratio < 0). (**b**) Genome-wide CNV profiles for non-cancer controls, gallbladder cancer (GBC), and non-GBC patients in the training cohort, shown as log2 ratio curves across autosomes. Red indicates copy number gain and blue indicates copy number loss.


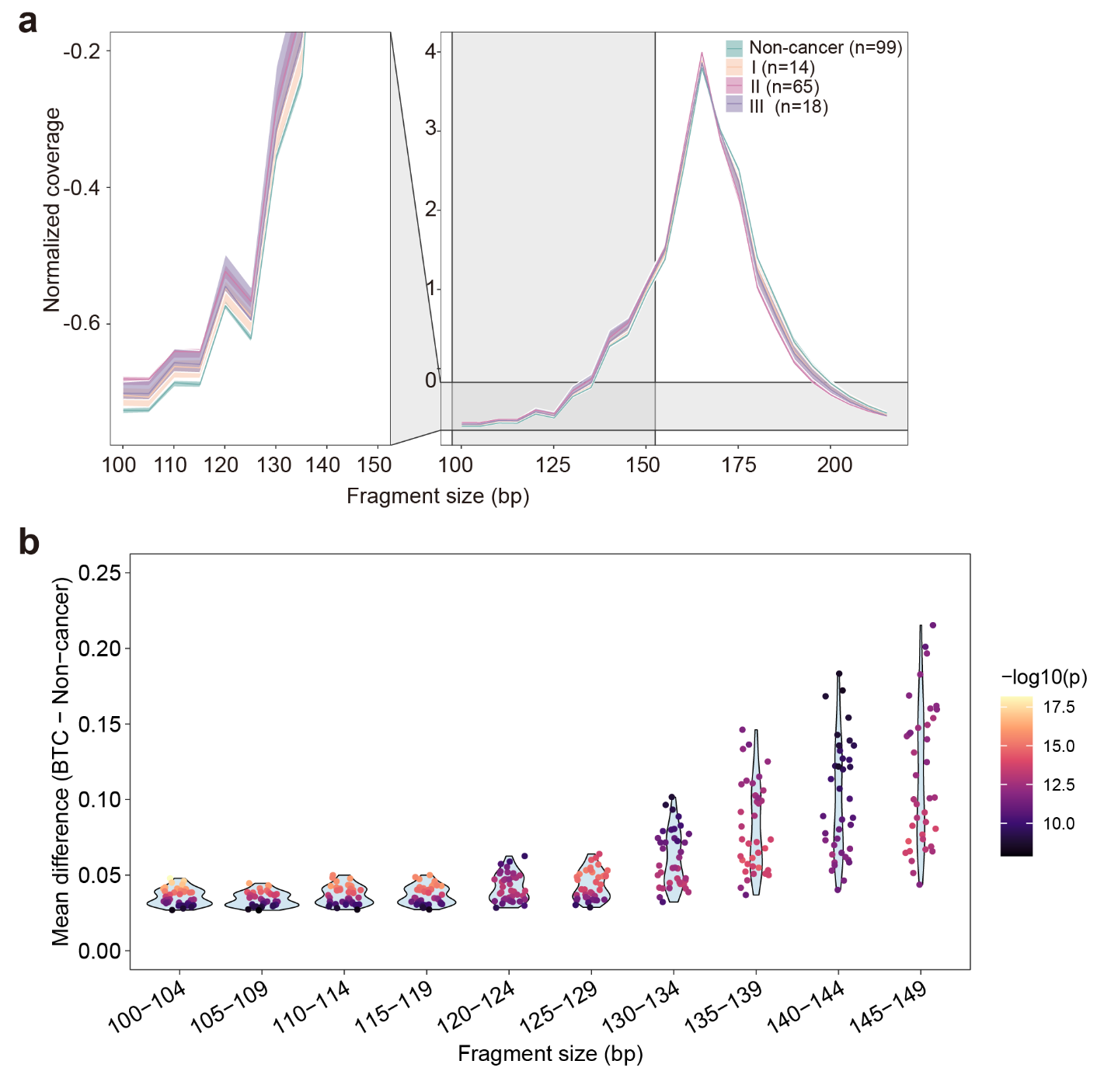


### **Figure S2. Fragment size distribution profiles**

(**a**) Fragment size distribution (FSD) patterns in BTC patients stratified by clinical stage and in non-cancer controls in the training cohort. (**b**) Violin plots of mean differences in 100–150 bp cfDNA fragments between BTC and non-cancer samples across chromosome arms. Each point is an arm, colored by Wilcoxon p-value.


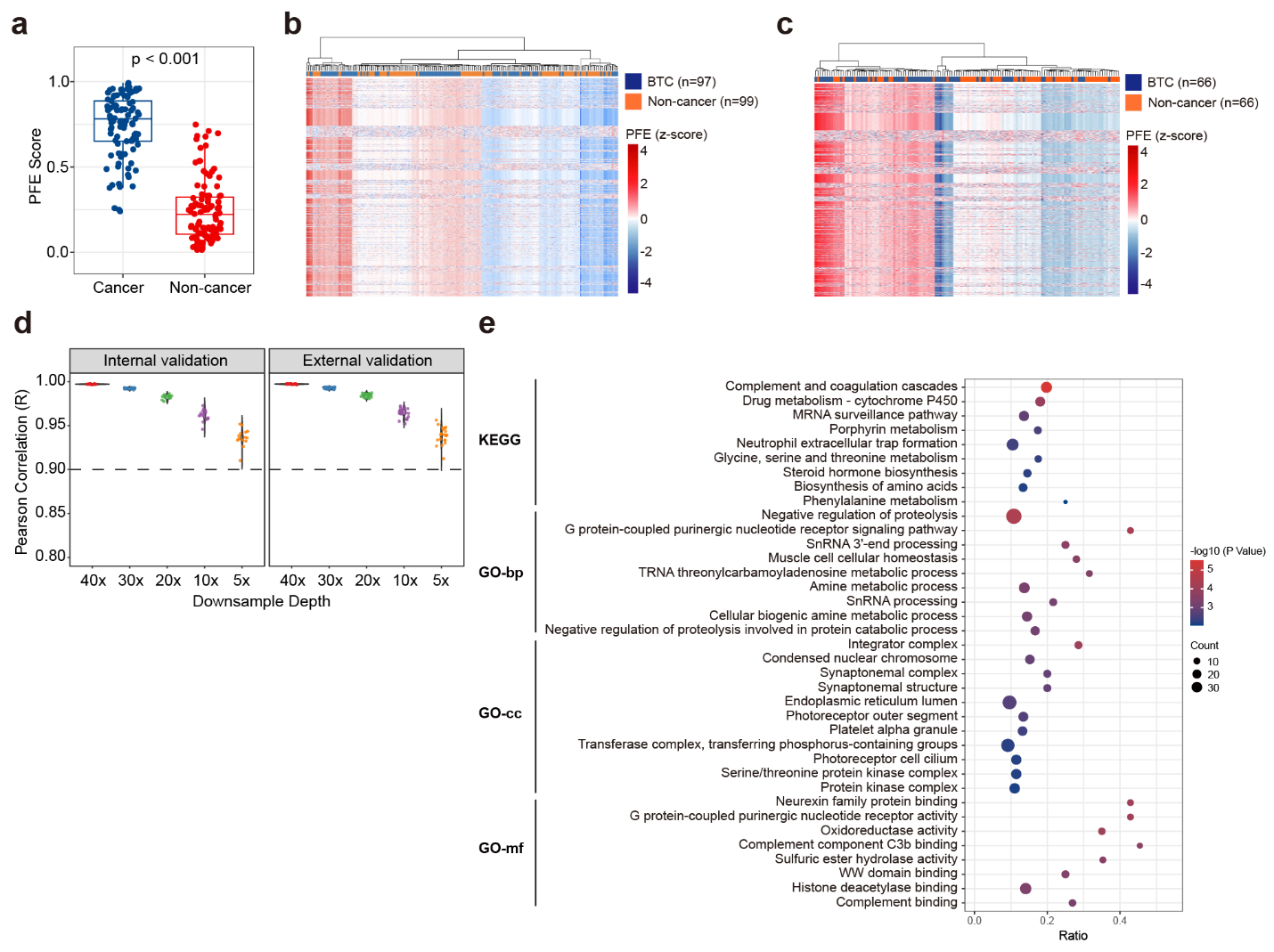


### **Figure S3. Promoter fragmentation entropy profiles**

(**a**) Box plot showing the distribution of PFE scores in cancer versus non-cancer participants in the training cohort. The p-value was calculated using the Wilcoxon rank-sum test. (**b, c**) Heatmap displaying normalized PFE values for the 1,203 selected PFE features in the training cohort (b) and the internal validation cohort (c). (**d**) Pearson correlation coefficients comparing PFE profiles derived from 50x reference data and lower-depth sequencing across validation cohorts. (**e**) KEGG and GO enrichment analyses of the 1,203 genes exhibiting significantly higher PFE values in BTC compared with non-cancer controls.


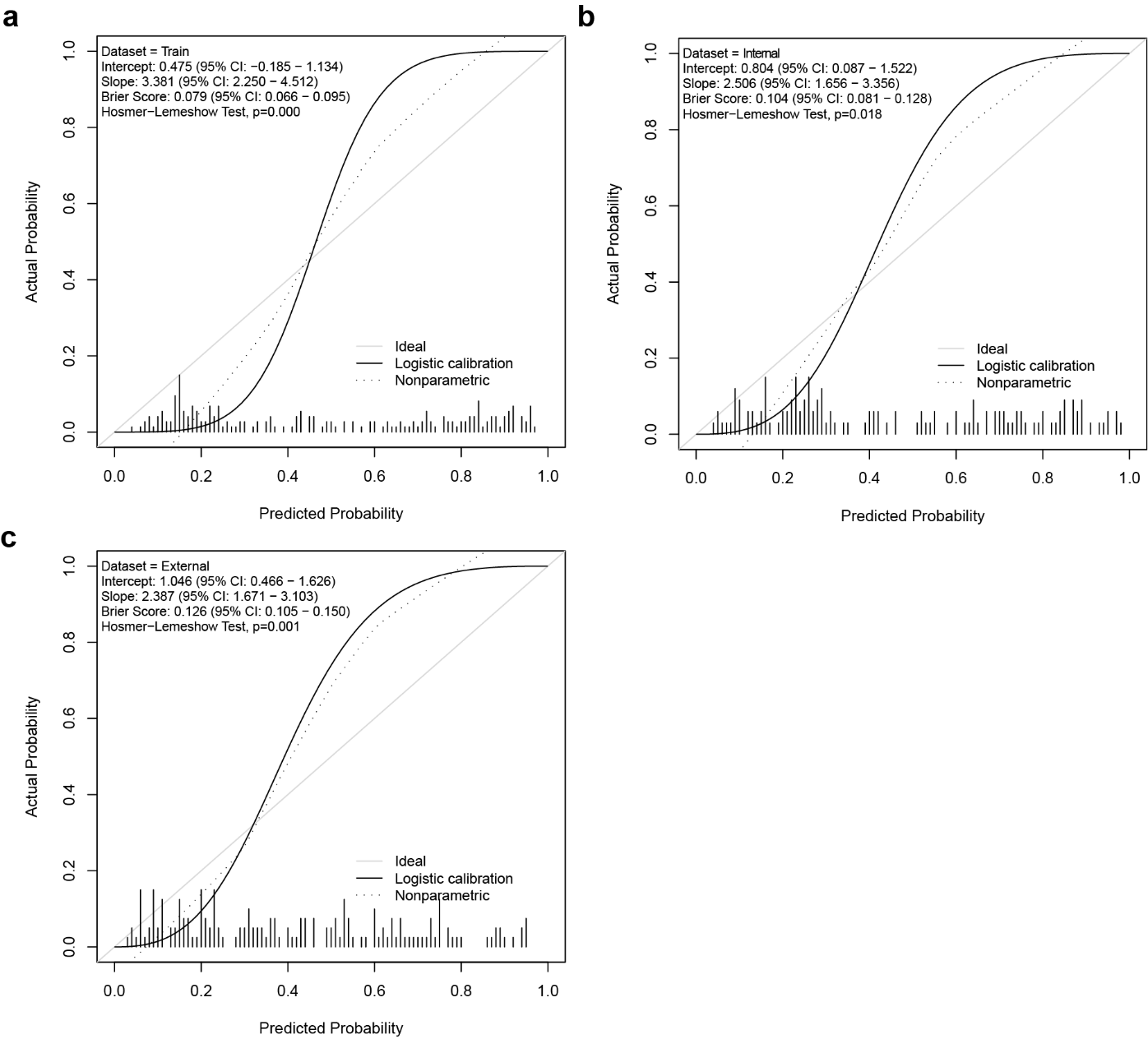


### **Figure S4. Probability calibration of the BTC risk score across cohorts**

Calibration plots are shown for the training cohort (**a**), internal validation cohort (**b**), and external validation cohort (**c**). The dashed diagonal line indicates ideal calibration (perfect agreement between predicted and observed probabilities). The solid line shows the logistic calibration curve obtained by regressing the observed outcome on the model-predicted log-odds, while the dotted line represents a nonparametric smoothed calibration curve. Rug marks along the x-axis indicate the distribution of predicted probabilities. Calibration intercept and slope, Brier score, and the Hosmer–Lemeshow test P value are reported within each panel.


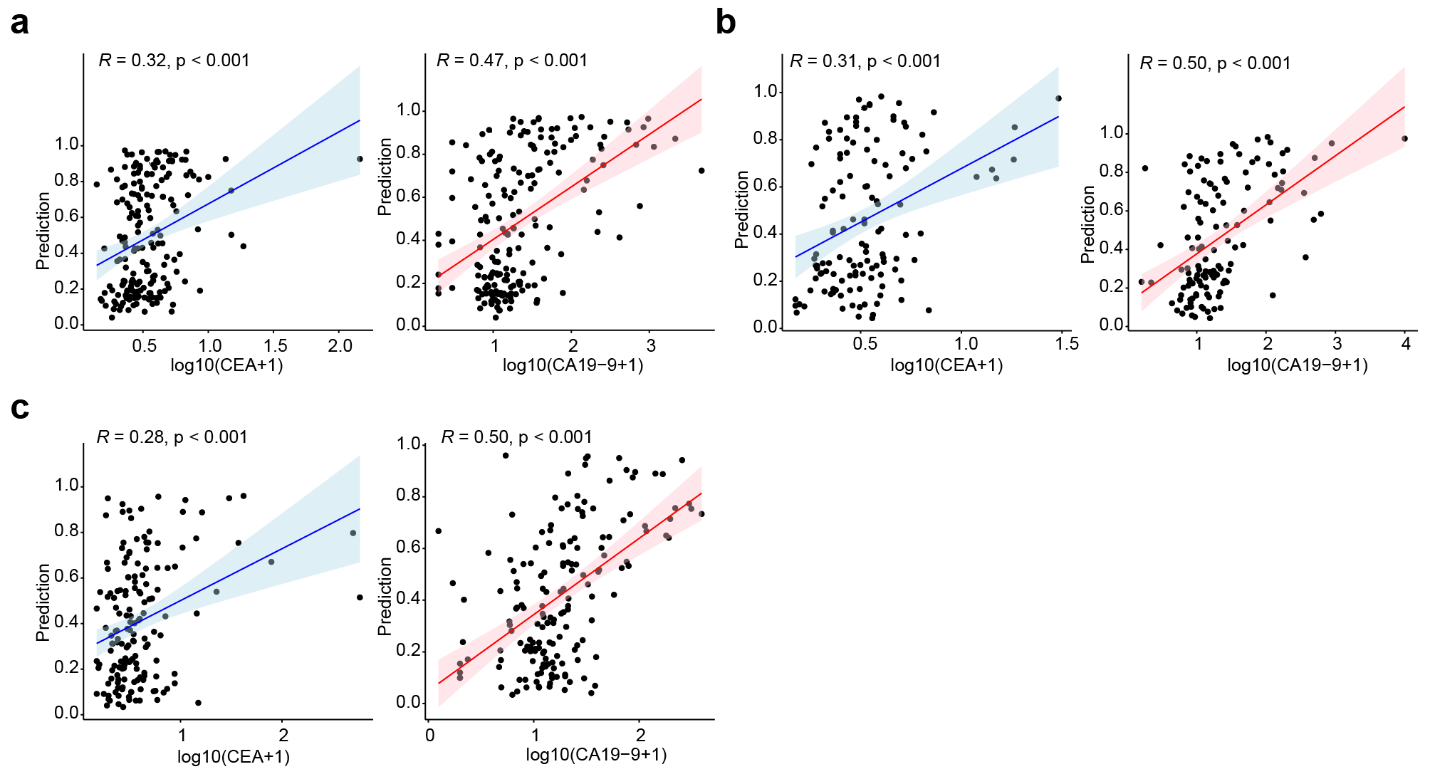


### **Figure S5. Correlation between cfDNA-based prediction scores and serum tumor markers**

Scatter plots show the relationship between model-derived prediction scores and log-transformed CEA (left) or CA19-9 (right) levels across the training (**a**), internal validation (**b**), and external validation (**c**) cohorts. Spearman correlation coefficients (*R*) and p-values are indicated in each panel.


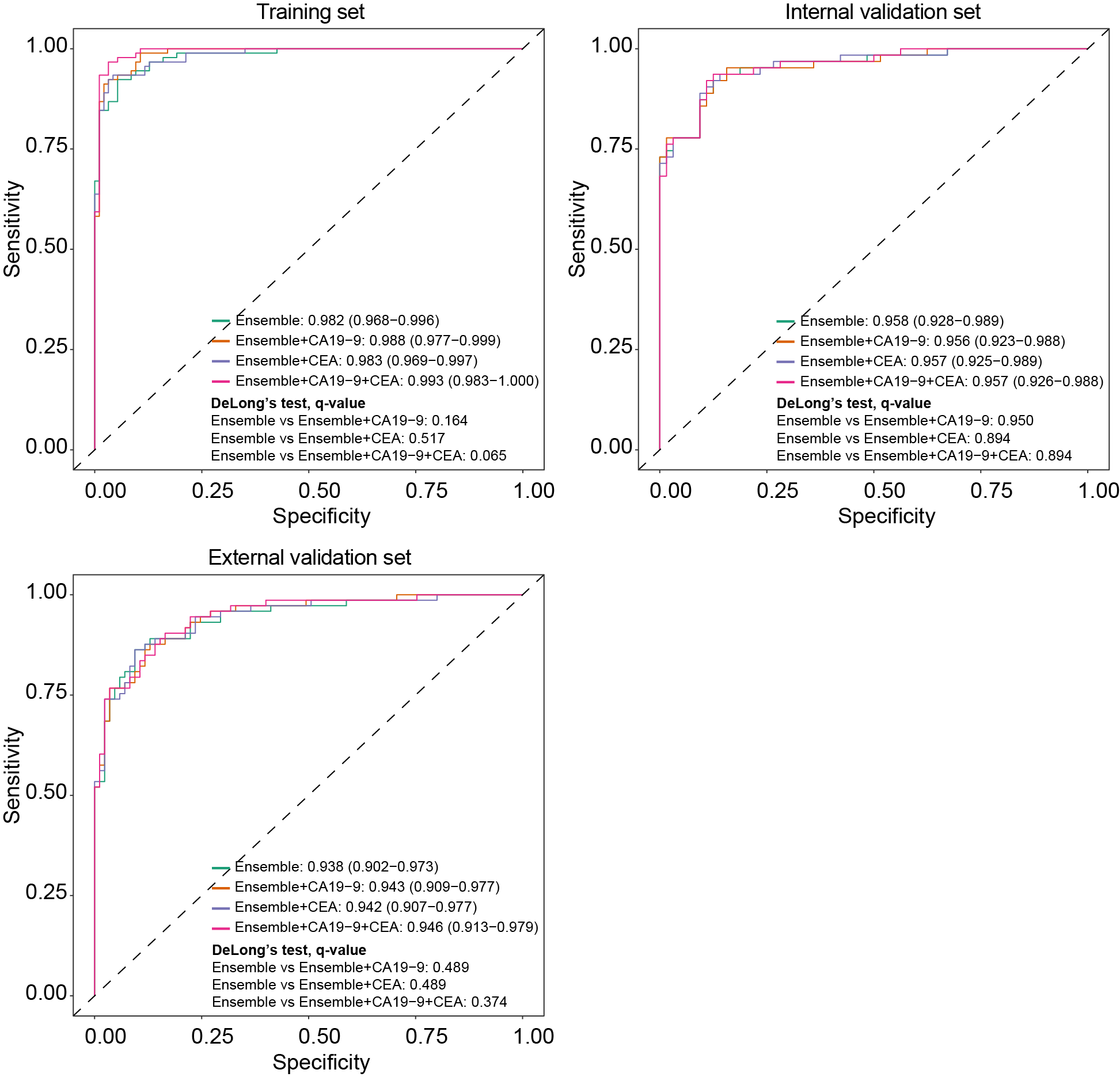


### **Figure S6. ROC curves comparing the fragmentomics-based ensemble model alone and in combination with serum biomarkers (CEA, CA19-9) across datasets**


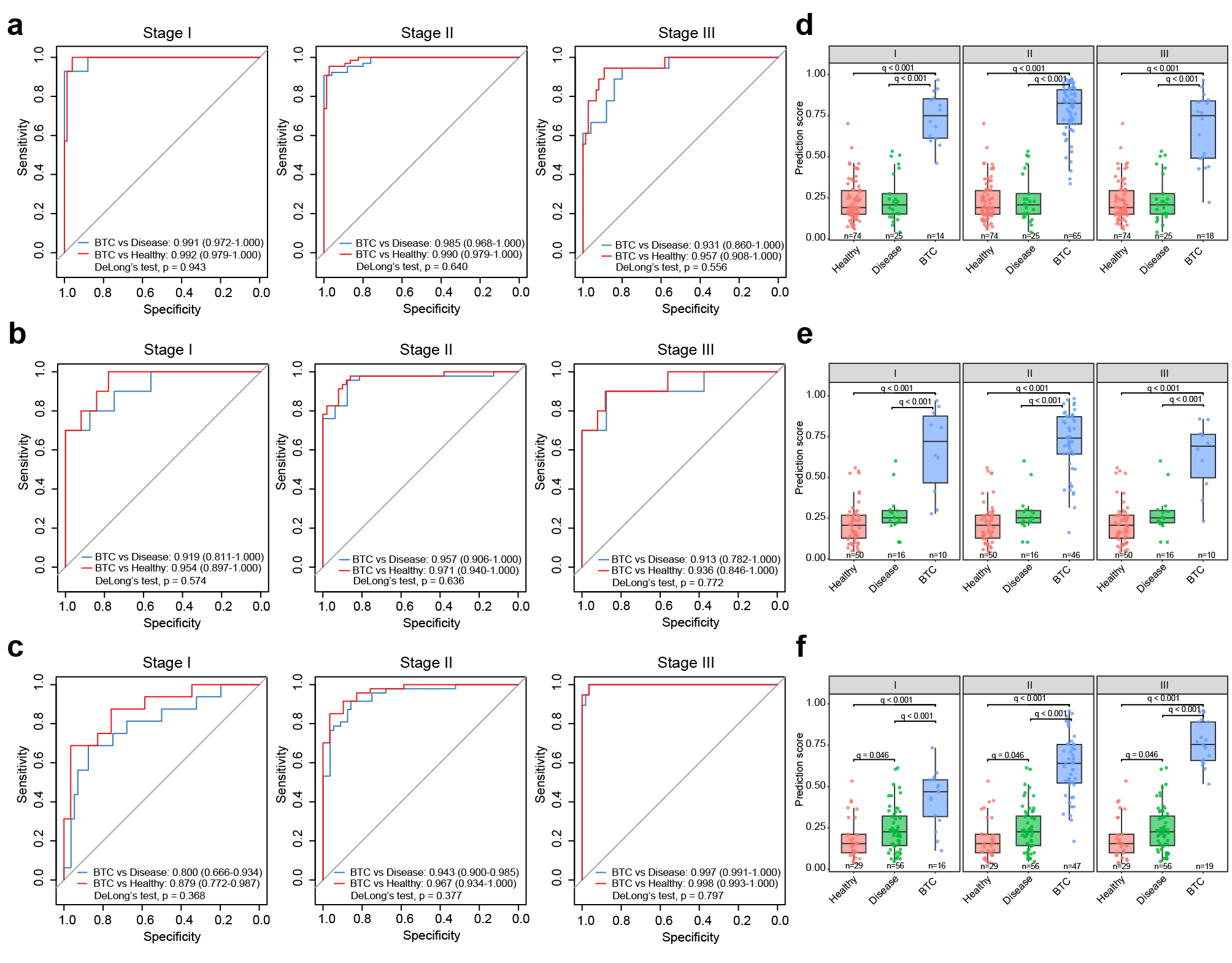


### **Figure S7. Model performance across clinical stages and non-cancer controls**

(**a-c**) Receiver operating characteristic (ROC) curves for BTC versus benign disease controls or healthy controls, stratified by clinical stage in the training (a), internal validation (b), and external validation (c) cohorts. (**d-f**) Prediction score distributions for healthy, benign disease, and BTC samples stratified by stage in the training (d), internal validation (e), and external validation (f) cohorts. Wilcoxon tests were used to compare groups, and p-values were FDR-adjusted.


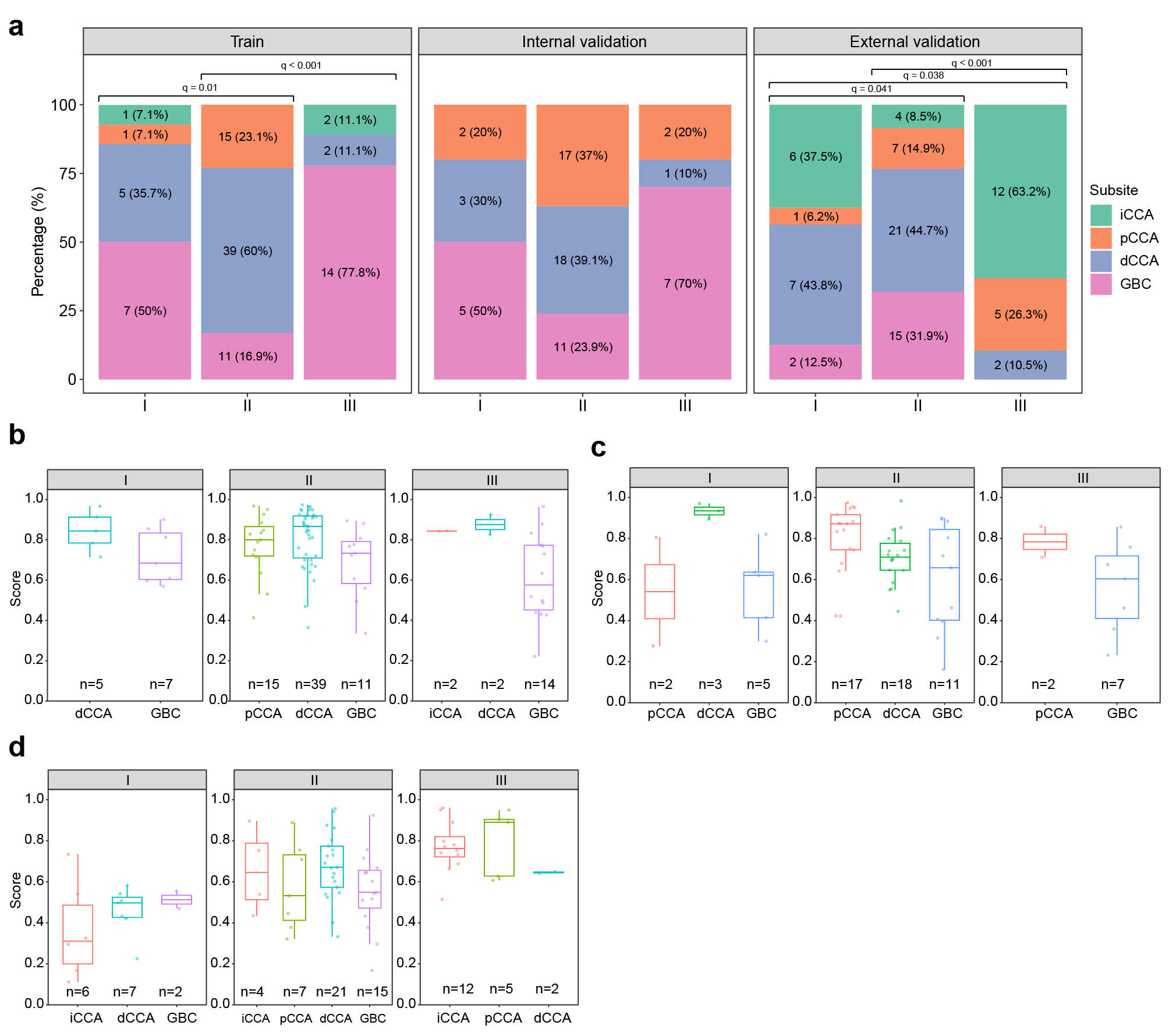


### **Figure S8. Stage-stratified analyses of risk scores between BTC subsites across cohorts**

(**a**) Stacked bar plots showing the distribution of BTC subsites within each clinical stage (I–III) across datasets. (**b–d**) Stage-stratified comparisons of predicted risk scores across BTC subsites.


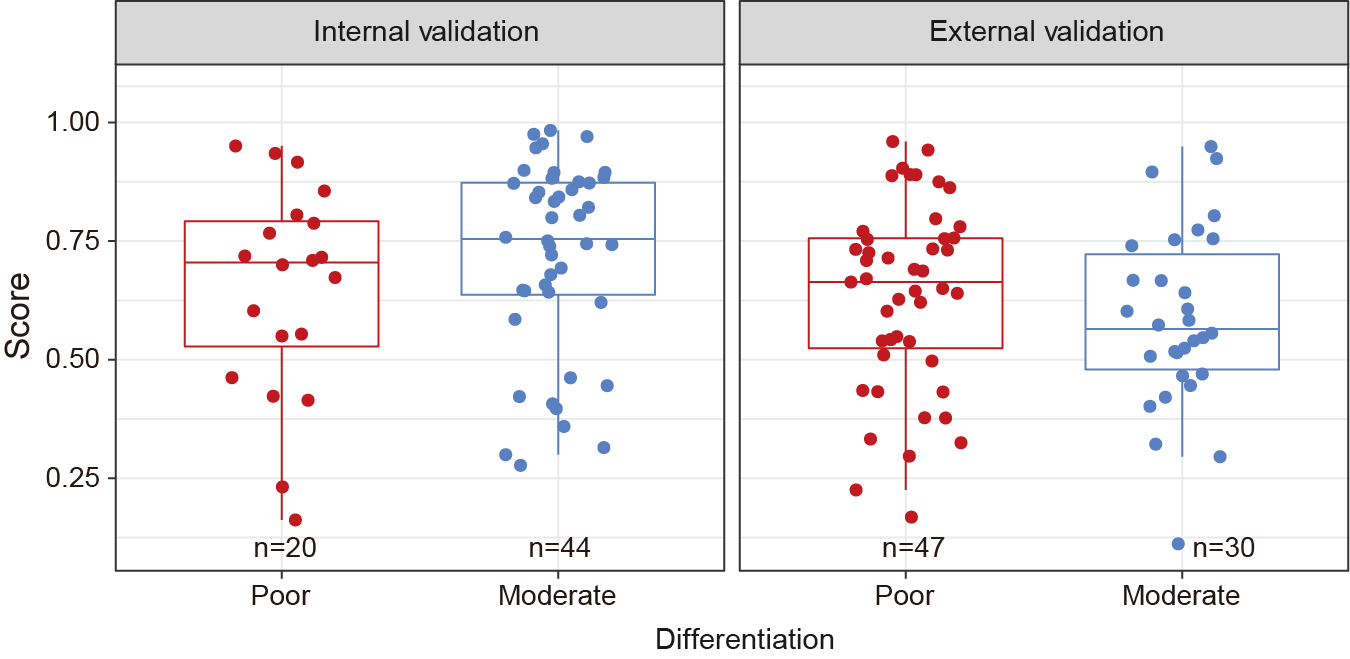


### **Figure S9. Distribution of BTC risk scores by tumor differentiation status**


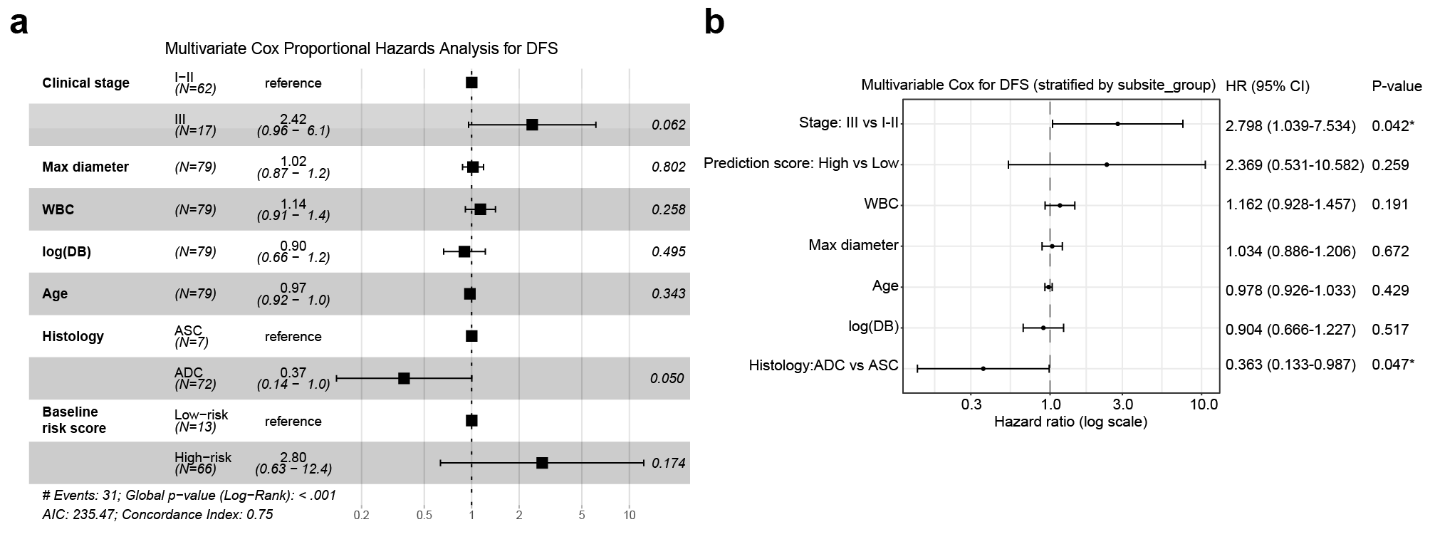


### **Figure S10. Baseline multivariate Cox model for disease-free survival**

(**a**) Forest plot showing hazard ratios (HRs) with 95% confidence intervals (CIs) for key clinical covariates and baseline risk scores in multivariate Cox regression analysis. (**b**) Subsite-stratified multivariate Cox analysis evaluating the prognostic association of the baseline risk score across BTC subsites.

Abbreviations: DFS, disease-free survival; WBC, white blood cell count; DB, direct bilirubin; GBC, gallbladder cancer; ADC, adenocarcinoma; ASC, adenosquamous carcinoma.


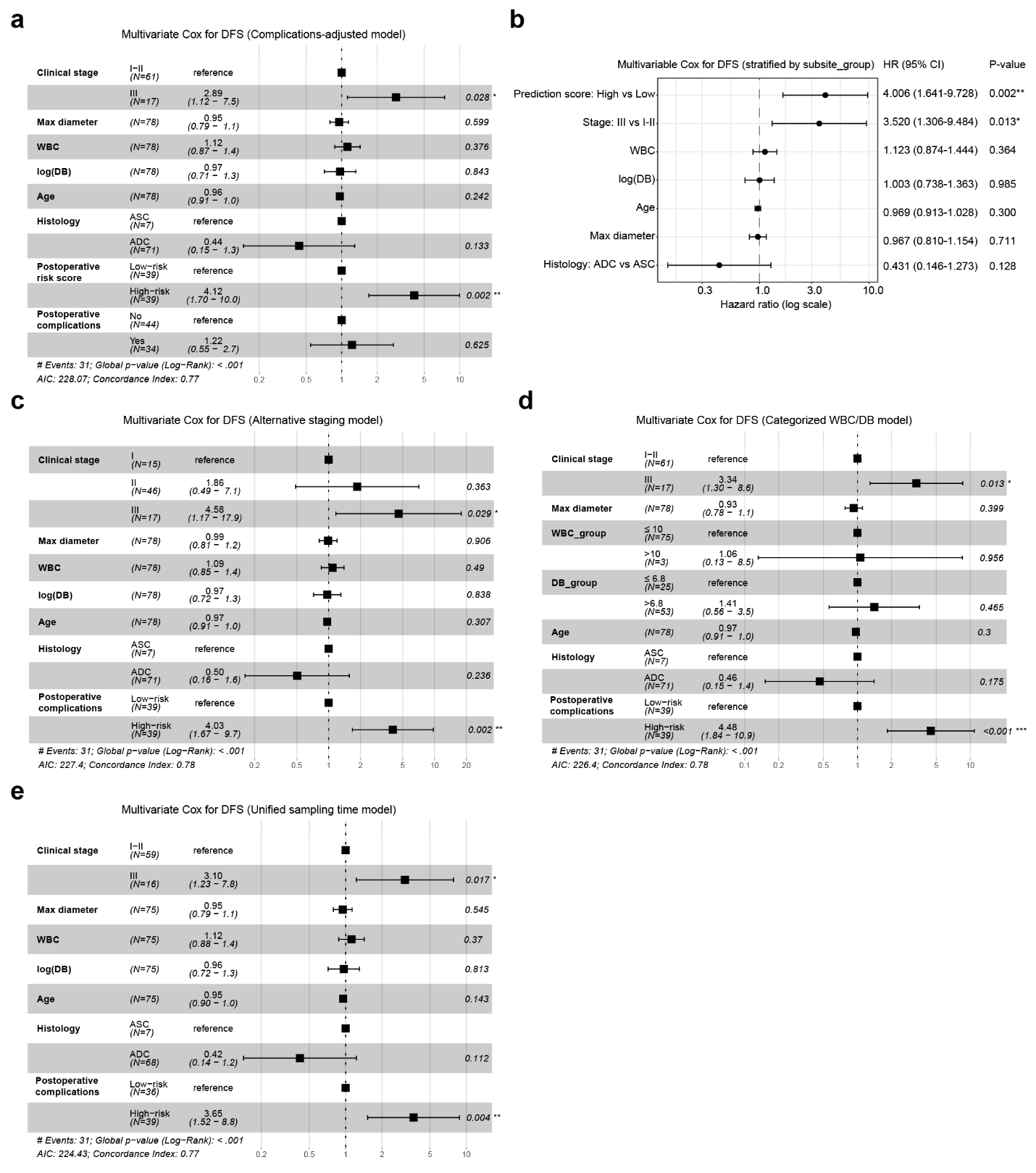


### **Figure S11. Sensitivity analyses of postoperative BTC risk score in the multivariate Cox model for disease-free survival**

Forest plot showing hazard ratios (HRs) with 95% confidence intervals (CIs) based on the primary model (shown in Figure 4e) with the following modifications: (**a**) additionally adjusted for postoperative complications; (**b**) subsite controlled using a stratified Cox model by subsite_group (GBC vs non-GBC) due to non-proportional hazards when modeled as a covariate; (**c**) stage modeled as three categories (I/II/III); (**d**) WBC and DB modeled as categorical variables; and (**e**) analysis restricted to patients sampled within 1 week after surgery (n=75).

Abbreviations: DFS, disease-free survival; HR, hazard ratio; CI, confidence interval; WBC, white blood cell count; DB, direct bilirubin; GBC, gallbladder cancer; ADC, adenocarcinoma; ASC, adenosquamous carcinoma.
